# Migraine is a dysfunction of neuronal potassium ion channels

**DOI:** 10.3389/fneur.2025.1622994

**Published:** 2025-07-31

**Authors:** Girishwaran M., S. Sajitha Lulu

**Affiliations:** Integrative Multiomics Laboratory, Department of Biotechnology, School of Bio Sciences and Technology, Vellore Institute of Technology, Vellore, India

**Keywords:** migraine, potassium channels, druggable targets, glibenclamide, levcromakalim, acrylamide (S-1)

## Abstract

Migraine is a primary headache disorder characterized by unilateral pain usually with aura, that affects approximately one in six individuals in India. The underlying biomechanical processes of migraine are still poorly understood, and new research is constantly being published. One of the major factors in migraine pathogenesis is the dysfunction of ion channels in the trigeminal nuclei and sensory cortices. Potassium channels are modulators and regulators of neuronal signaling and conductance, playing an important role in maintenance of the membrane potential and neuronal conduction. Therefore, potassium channel dysfunctions are potential factors in migraine pathogenesis, and thus targets for specific antimigraine prophylaxis. This review reveals that potassium channels play a significant role in pathogenesis and management of migraine. Dysfunctions in K_ATP_ channels, K_2P_ channels including TRESK and TREK-1, small and large conductance calcium-sensitive potassium channels (SK_Ca_ and BK_Ca_), and voltage-gated potassium channels (K_V_) are known to affect the incidence and progression of migraine in the general populace. K_ATP_ openers can induce migraine like phenotype, but K_ATP_ blockers have so far not been effective in reducing the intensity of migraine headache. Potassium channels are a potential druggable target for migraine prophylaxis with several compounds currently in preclinical trials.

## 1 Introduction

Migraine can be defined as an recurrent headache disorder (1), and is diagnosed clinically based on criteria outlined in ICHD–3 (2). It is one of eleven primary headache disorders (3). It consists of unilateral acute or chronic period headaches with some presentation of nausea and/or light, sound, or smell sensitivity (1, 4). For a while, migraine was considered as a primarily vascular disorder with neurological symptoms emerging as epiphenomenons due to vascular changes; however, that theory is no longer considered viable (5–7). Vasodilation or constriction is instead thought to be an emergent symptom as a result of instability in neuronal control mechanisms (8).

Migraine presents in four stages that each occur sequentially from or simultaneously with the previous: premonitory symptoms or prodrome, aura, headache, and postdrome (1, 4). Aura may not be apparent in all cases of migraine.

The genetic or inheritable factors influencing migraine are varied and poorly understood. Several genes have been implicated in familial hemiplegic migraine, and in chronic and acute migraine, both nuclear and mitochondrial in origin (9–12). The exact functional impact of these genes in predisposition to migraine is not yet fully elucidated, but several theories have been put forth (9–12).

## 2 Mechanisms underlying migraine symptoms

**Figure 1:**
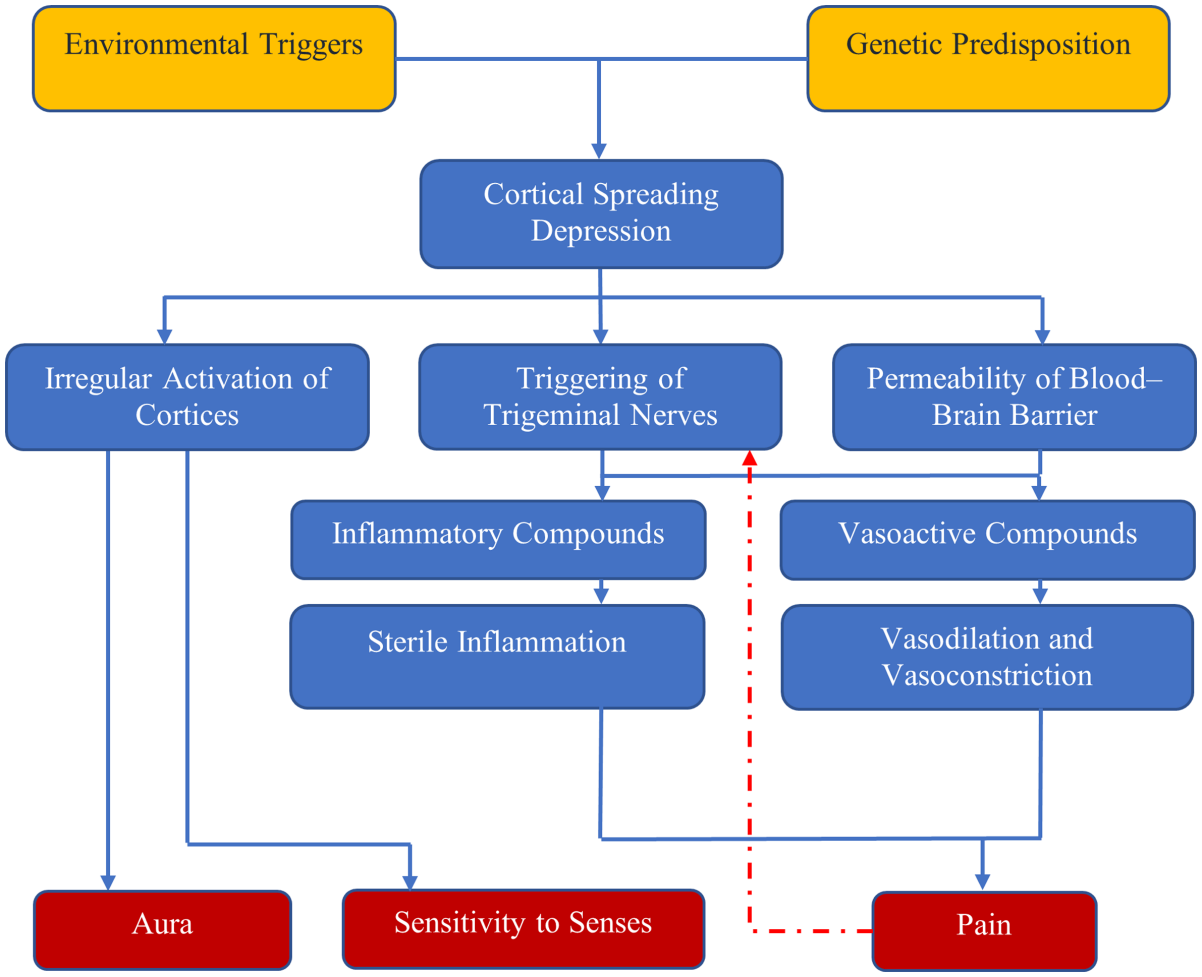
Mechanisms of migraine—environmental triggers and genetic predisposition result in cortical spreading depression, which has three effects: irregular activation of cortices, which results in aura and sensitivity to senses; and triggering of trigeminal nerves and increased permeability of blood–brain barrier, which result in pain due to vasodilation and sterile inflammation.

The current understanding of the mechanisms underlying migraine is as follows:

A dysfunction of neuronal polarization in the brain results in a sequence of intracranial and extracranial changes that result in migraine symptoms (1, 4). Figure 1 shows the currently accepted mechanism behind migraine attacks.

### 2.1 Cortical spreading depression

The phenomenon known as cortical spreading depression (CSD), first described by Leão in 1944 (13, 14), is the currently accepted physiological phenomenon that causes both headaches and aura in migraine (5). CSD is a wave of depolarization that spreads across the nerves and glial cells in the cerebral cortex in a self-propagating manner. CSD has been theorized to:

1. Directly cause the aura in migraine through abnormal activation of neurons in the sensory cortices (15).
2. Induce altered nociception by activating trigeminal nerve afferents (16, 17), and
3. Alter the permeability of the blood–brain barrier (18).

The activation of afferent nerves to the trigeminal nerves, resulting in inflammatory changes in pain-sensitive regions of the meninges, causes the headache of migraine (18). A molecular cascade of events—likely involving pannexin-1, capsase-1, and several proinflammatory mediators (16, 17)—is the likely pathway linking CSD to the prolonged activation of trigeminal nociception that is the generating source of the pain of migraine. Migraine without aura might be linked to CSD in regions of the brain where the depolarization does not result in sensory alteration (19).

### 2.2 The trigeminovascular system

The trigeminovascular system is a primarily nociceptive system of neurons consisting of several small caliber pseudo-unipolar sensory neurons that originate from the trigeminal ganglion and cervical dorsal roots (20), before spreading to innervate cerebral vessels, vessels in the pia and dura matters, and venous sinuses. The nerves from the cervical roots and the trigeminal ganglion converge at the trigeminal nucleus caudalis (21, 22). The trigeminovascular system also receives signals from a vast array of regions in the brain and brain-stem (20, 23–32) and transmits nociceptive signals to the limbic system for the emotional and vegetative processes following pain (30).

The activation of the trigeminovascular system either by physical pain signals or CSD results in the release of vasoactive compounds including Substance P, calcitonin gene related peptide (CGRP), and Neurokinin A (33). These result in vasodilation and neurogenic inflammation, which are both important in the prolongation and intensification of pain from migraine (34).

## 3 Review methodology

Articles were retrieved from PubMed and SCOPUS in a manner respecting the PRISMA–2020 guidelines using the following search criteria (exact text was modified for SCOPUS to adhere to the functional words in the SCOPUS search engine):

1. (potassium channels) AND (migraine)
2. ((potassium channels) AND (migraine)) AND (treatment)
3. (potassium channels) AND (channelopathies)
4. (voltage gated potassium channels) AND (channelopathies)
5. (calcium gated potassium channels) AND (channelopathies)
6. (ATP sensitive potassium channels) AND (channelopathies)
7. (two-pore domain gated potassium channels) AND (channelopathies)
8. (inward rectifying potassium channels) AND (channelopathies)

Bulk deduplication was done using a deduplicator plugin for Zotero, and the results manually curated.

The following screening, inclusion, and exclusion criteria were used to refine the results:

1. Screening criteria:

a) DOI leads to empty or broken website
b) source type is correspondence, editorial, operative editorial, or other form of non-rigorous writing
c) article not in English AND English translation cannot be acquired or generated

2. Inclusion criteria:

a) Discusses potassium channels with respect to migraine

3. Exclusion criteria:

a) Article focused on disease other than migraine
b) Article focused on molecule other than potassium channel protein

**Figure 2:**
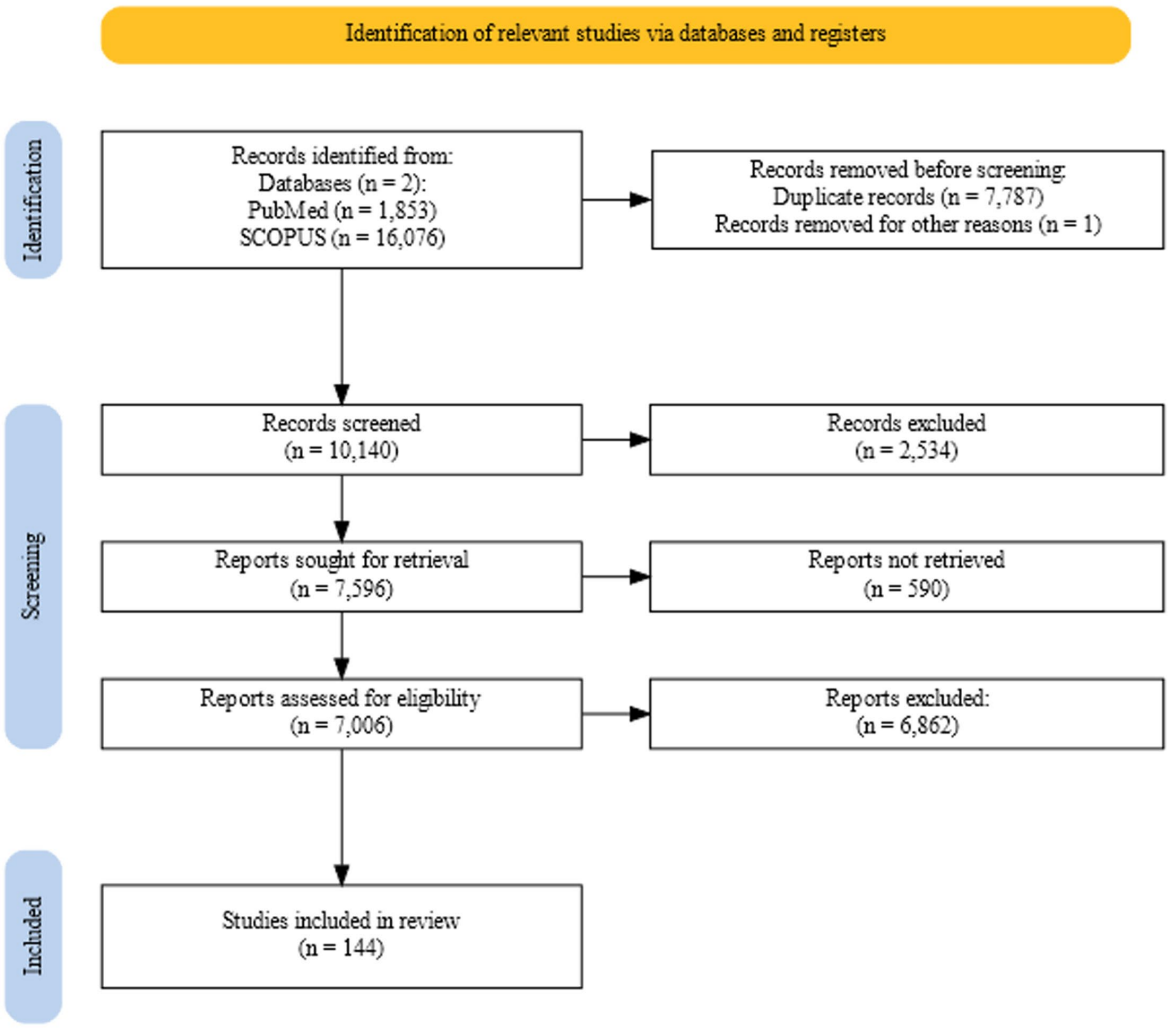
Flowchart showing the retrieval, screening, and assessment process for the review generated using the web tool PRISMA2020 (183).

Figure 2 shows retrieval, screening, and assessment process for the review. Supplementary Table 1 holds the final set of included articles with the information contained relevant to the review.

## 4 Ion channels

Ion channels are pore-forming transmembrane proteins that facilitate the transfer of ions across the cell membrane. Ion channels are found in most cells across various tissues, however the ion channels implicated in migraine are those found in neural tissues and the endothelial, epithelial, or smooth muscle cells of the vascular tissues (35–44). As ion channels are essential agents in the creation and propagation of potential across the neuronal cells, it is no surprise that several ion channel proteins have been implicated in hereditary forms of migraine. The voltage-gated calcium channel coded by members of the *CACNA1* family of genes, the sodium-potassium ATPase pumps from the *ATP1A* family of genes, and the voltage-gated sodium channel coded by the *SCN1A* gene have been long associated with familial hemiplegic migraine (40, 43–59). However, these same channels do not seem to be associated with non-hemiplegic migraine (46), and therefore there must be a different channel or channels that are involved more closely with these types of migraine.

### 4.1 Potassium channels

Potassium channels are regulatory channels that control cell potential, and the secretion of hormones and neurotransmitters, and modulate the frequency and shape of the action potential waveform both in the peripheral and central nervous systems. They are regulated in turn by either transmembrane voltage, calcium ions, or neurotransmitters, or by the very signal pathways they stimulate. Potassium channels are classified into three families, depending on the number of transmembrane domains in the protein structure: Two Trans-Membrane Domains (2TM), Four Trans-Membrane Domains (4TM), and Six Trans-Membrane Domains (6TM) (39).

#### 4.1.1 2TM channels

**Figure 3:**
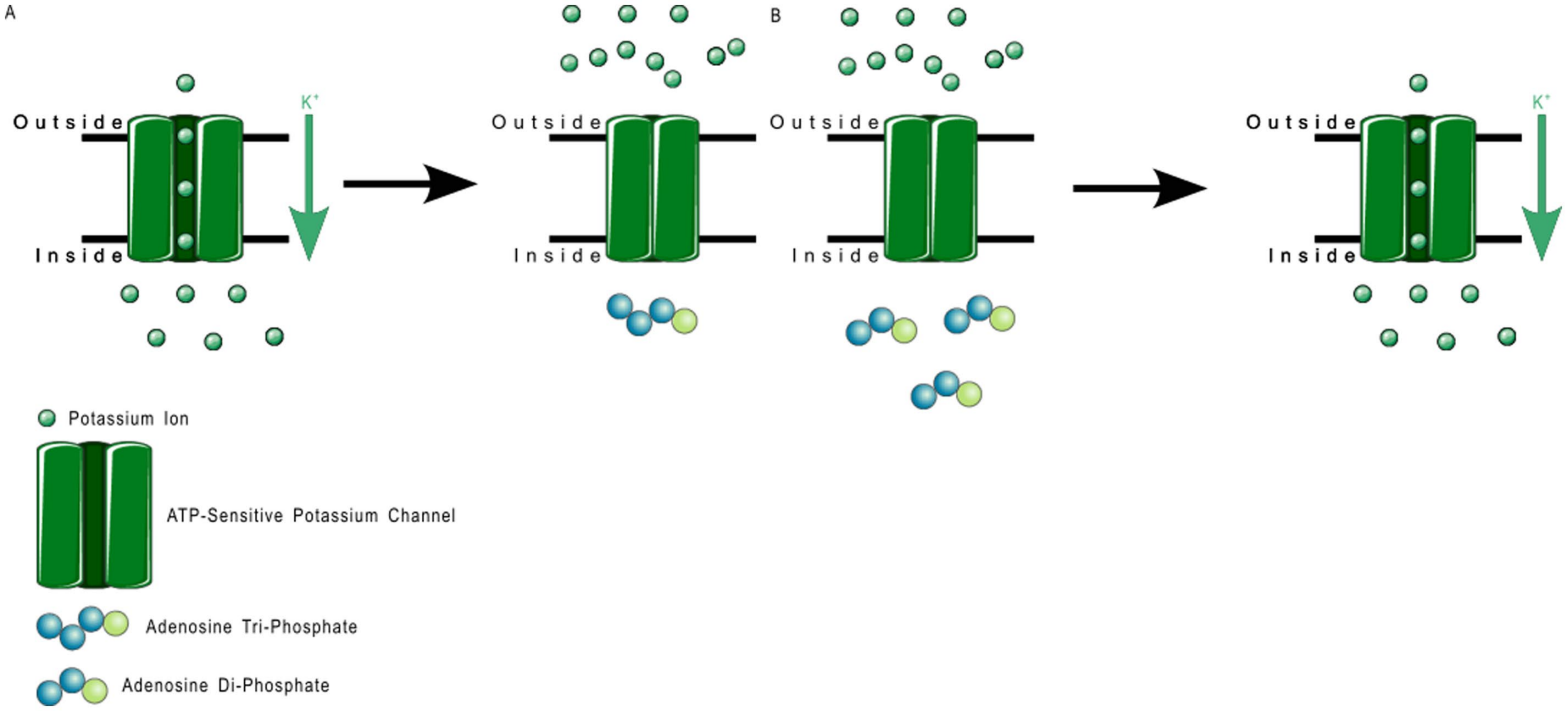
ATP-sensitive KIR6.1 function. **(A)** Depicts the deactivation trigger: the *presence* of adenosine triphosphate near the cell interior region of the channel protein prevents potassium transport; and **(B)** depicts the activation trigger: the *absence* of adenosine diphosphate near the cell interior region of the channel protein allows potassium transport.

2TM channels are inward rectifying channels (K_IR_) responsible for controlling the rate of depolarization and repolarization. They play important roles in the functioning of various organs including the brain, heart, liver, kidney, skeletal muscle, pancreas, and retina (39, 60–66). K_IR_ channels are usually constitutively active, but two subfamilies are activated by specific proteins or cytosolic concentrations of certain compounds (39, 60). K_IR_ channels are described in more detail in Table 1. Figure 3 shows the normal functioning of KIR6.1, where (Figure 3B) increasing ADP concentration opens the channel but (Figure 3A) even a small concentration of ATP near the channel closes it.

**Table 1:** Inward rectifying potassium channels—data taken from several sources (39, 60–66, 116).

Subfamily	K_IR_6.x	
Type	K_IR_6.1	K_IR_6.2
Function	ATP Sensitive Inward Rectifying Current	ATP Sensitive Inward Rectifying Current
Activation	Increased concentration of nucleoside diphosphates (NDPs) (ADP, GDP, etc.)	Magnesium-bound ADP
Inactivation	ATP	ATP
Activators	NDPs, diazoxide, pinacidil, nicorandil	MgADP, diazoxide, pinacidil, cromakalim, nicorandil
Inhibitors	None	None
Gating Inhibitors	None	ATP
Blockers	Glibenclamide	Sulfonylurea compounds, benzamide derivatives, glinides
Organ(s)	Vascular smooth muscle	Pancreatic β cells, heart, skeletal muscle, brain
Channelopathies	Suspected in vasospastic angina, migraine	Implicated in Persistent Hyper-insulinemic Hypoglycemia of Infancy (PHHI), certain forms of diabetes, migraine

**Figure 4:**
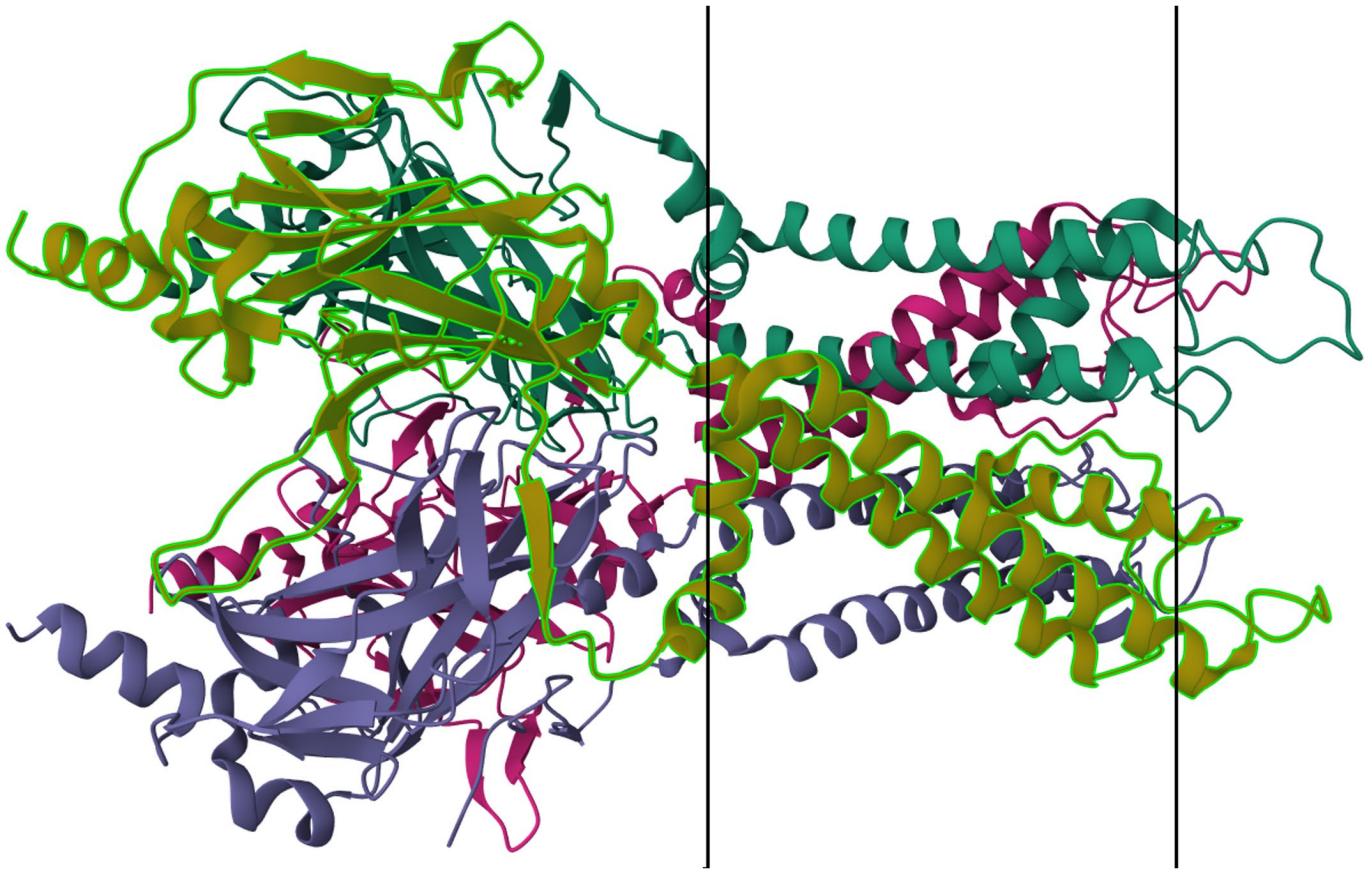
Protein model of KIR6.2 tetramer. The molecule is oriented such that the cytosolic region falls to the left of the manuscript. The two surfaces of the cell membrane are denoted by two black bars. Image modified from Martin et al. (184) as retrieved from the RCSB Protein Data Bank.

Members of the K_IR_6.x subfamily are ATP-sensitive, and are implicated in the pathology of several primary headache disorders, including migraine (67, 68). Kubo et al. (60) in their 2005 publication refer to two articles that describe mutations in K_IR_4.1 that confer resistance or susceptibility to hyperexcitability. Al-Karagholi and Hansen et al. —in their 2019 study of 16 patients—implicate K_IR_6 in migraine onset, using response to levcromakalim, a potent K_IR_6 blocker (69). Al-Karagholi et al., later in 2019, however, show that levcromakalim does not cause migraine through affecting peripheral neurons (70). Al-Karagholi and Ghanizada et al. in 2021 repeated their study on the role of levcromakalim in the central nervous system (CNS) and showed again an association with migraine (71). Christensen et al. —in their 2020 publication—describe glibenclamide-induced reversal of cephalic hypersensitivity and CGRP release by interacting with regulatory subunits of KIR6.1 (72). Clement et al. —in their 2022 (73) and 2023 (74) reviews on the subject—strongly recommend further research into KIR6. 1 and 6.2 blockers as migraine prophylactics, based on findings in mouse models. Dyhring et al. in 2023 also recognize K_ATP_ blockers as potential migraine prophylactics, recommending K_IR_6.1/SUR2B as a potential drug target for discovery studies (75). Figure 4 shows a ribbon model of the K_IR_6.2 tetramer.

#### 4.1.2 4TM channels

**Figure 5:**
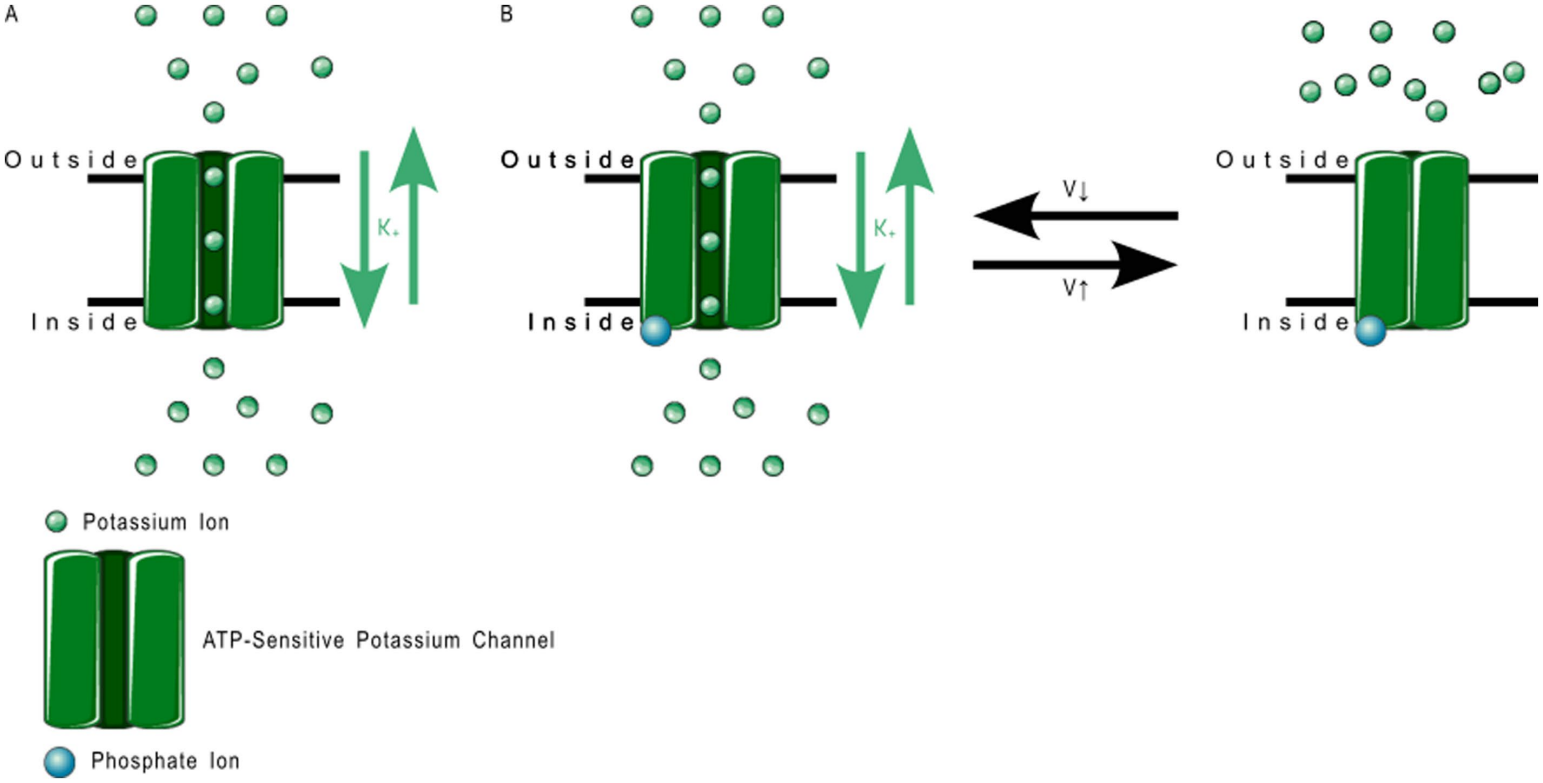
TREK-1 function. **(A)** Depicts the normal function: open rectification where the ions flow according to the charge gradient, whereas **(B)** depicts the altered function upon phosphorylation, where change in membrane voltage switches the channel between open and closed.

**Figure 6:**
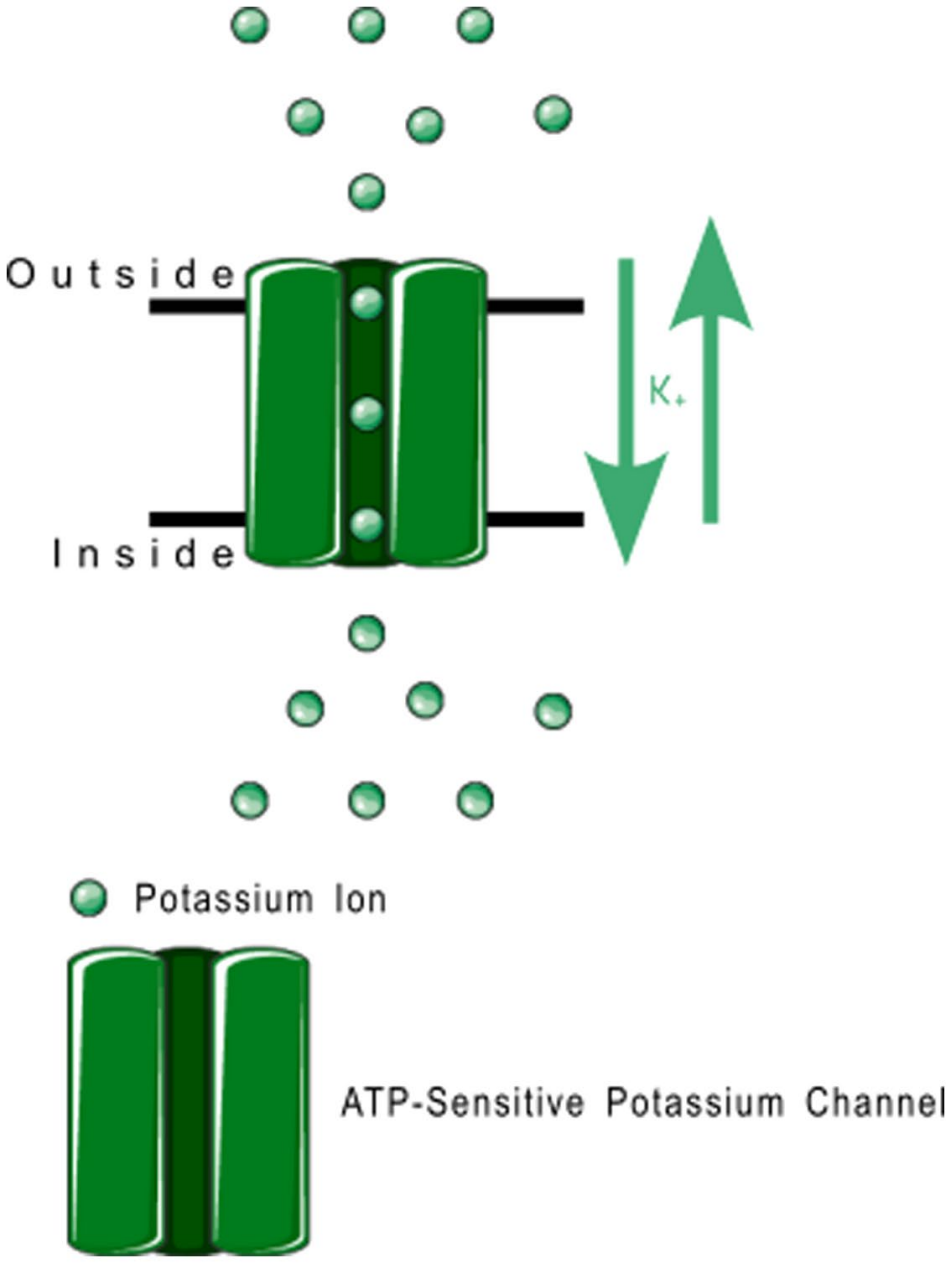
Open rectifier function. TRESK, TASK, TREK-2, and TRAAK are all open rectifiers, which allow free flow of potassium ions both ways across the plasma membrane.

4TM channels are tandem–pore-domain channels responsible for controlling leak currents and are constitutively active at all voltages. The type family is Tandem–Pore-Domain-Containing Weak Inward-Rectifying Potassium(K) channel 1 (TWIK-1 or K_2P_1.1) K_2P_ channels stabilize resting membrane potential and counteract depolarization (76–78). K_2P_ channels are described in more detail in Table 2. Figure 5 shows the Normal Function of TWIK-Related Potassium(K) Channel 1 (TREK-1 or K_2P_2.1): it is an open rectifier channel that allows potassium ions to move in or out of the cytosol to maintain homeostasis. However, as shown in B, TREK-1 can be phosphorylated to turn it into a voltage-gated rectifier instead, which allows ions through dependent on the local membrane potential. Figure 6 shows the general normal function of K2P channels. TREK-2 (K_2P_10.1), TWIK-Related Spinal-Cord Potassium(K) Channel (TRESK or K_2P_18.1), TWIK-Related Acid-Sensitive Potassium (K) Channel (TASK or K_2P_3.1, 5.1, and 9.1) and TWIK-Related Arachidonic-Acid Sensitive Potassium(K) Channel (TRAAK or K_2P_4.1) are always open rectifiers. TASK family channels are blocked — physiologically — when the pH inside the cell lowers below certain thresholds. TRAAK only activates when the temperature rises above 31° C. TRESK is dependent on intracellular calcium concentration.

**Table 2:** Two-pore doman potassium channels—data taken from several sources (39, 76, 79, 80, 118, 135, 138, 142, 188–191).

Subfamily	K_2P_18.x
Type	K_2P_18.1
Function	Open Rectifiers
Activation	Instantaneous calcium-dependent activation
Inactivation	Slow inactivation
Activators	Non-Cytoplasmic Ca^2+^, calcineurin, volatile anaesthesia
Inhibitors	None
Gating inhibitors	None
Blockers	Ba^2+^, quinine, quinidine, Extracellular acidification (pH cutoff unknown)
Organ(s)	Cerebrum, cerebellum, brain stem, spinal cord, testis
Channelopathies	Loss-of-function mutations found in migraine with and without aura patients, as well as familial hemiplegic migraine families

**Figure 7:**
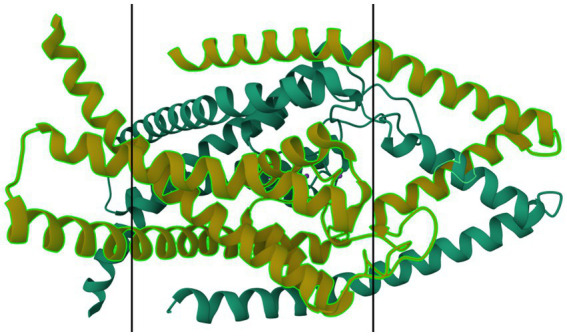
Protein model of TASK-2 channel (K2P5.1) dimer. The molecule is oriented such that the cytosolic region falls to the left of the manuscript. The two surfaces of the cell membrane are denoted by two black bars. Image modified from Li et al. (185) as retrieved from the RCSB Protein Data Bank.

Mutations in many subfamilies of the 4TM family, especially TRESK and TREK, are strongly associated with inheritable (familial) migraine with aura. Lafrenière et al. in 2010 discovered a frameshift mutation in migraine patients that was predicted through functional characterization to cause a complete lack of TRESK function (79). Lafrenière and Rouleau—in their 2011 review—discuss the role of TRESK channels in migraine, including a potential role of TRESK loss-of-function in migraine-like side effects of cyclosporin treatment (80). Royal et al. —in their English-language publication in 2019—discuss the role of a TRESK mutation (TRESK-MT) that causes loss-of-function of TRESK channels, associating this shift with primary headache disorders including migraine (81). Royal, with a different group of authors, also published a French-language review in the same year discussing the role of TRESK-MT and other loss-of-function TRESK mutations in migraine (82). Verkest et al. in their 2021 review discuss activators and inhibitors of TRESK, TREK-1, and TREK-2 as playing “a key role in […] neuronal excitability,” recommending these channels as targets for migraine treatment. Lengyel et al. in 2020 showed that this mutation produced a fragment that also interfered with TREK-2 (83). Ávalos Prado et al. published a focused review of TREK family channels and their role in headache and migraine. The paper describes increased neuronal excitability, especially in trigeminal neurons, as a triggering factor for migraine attacks caused by mutations in TREK family channels, especially TREK-1, TREK-2, and TRAAK (Twik-Related Arachidonic Acid-sensitive K^+^ channels) (84). Figure 7 shows a ribbon model of the TASK-2 dimer.

#### 4.1.3 6TM channels

**Figure 8:**
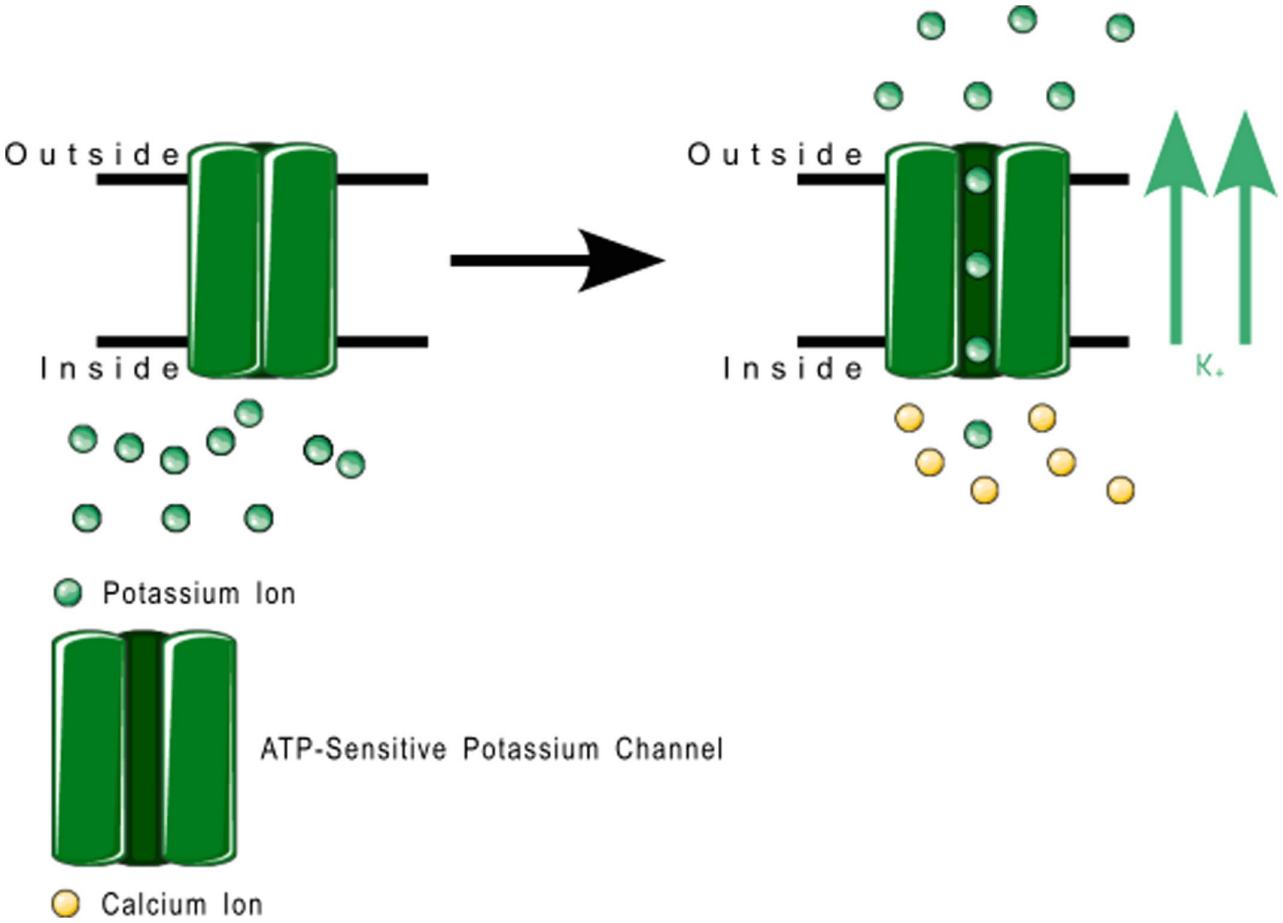
Large conductance calcium-gated potassium channel function. These channels regulate membrane depolarization by pushing potassium channels into the extracellular space.

**Figure 9:**
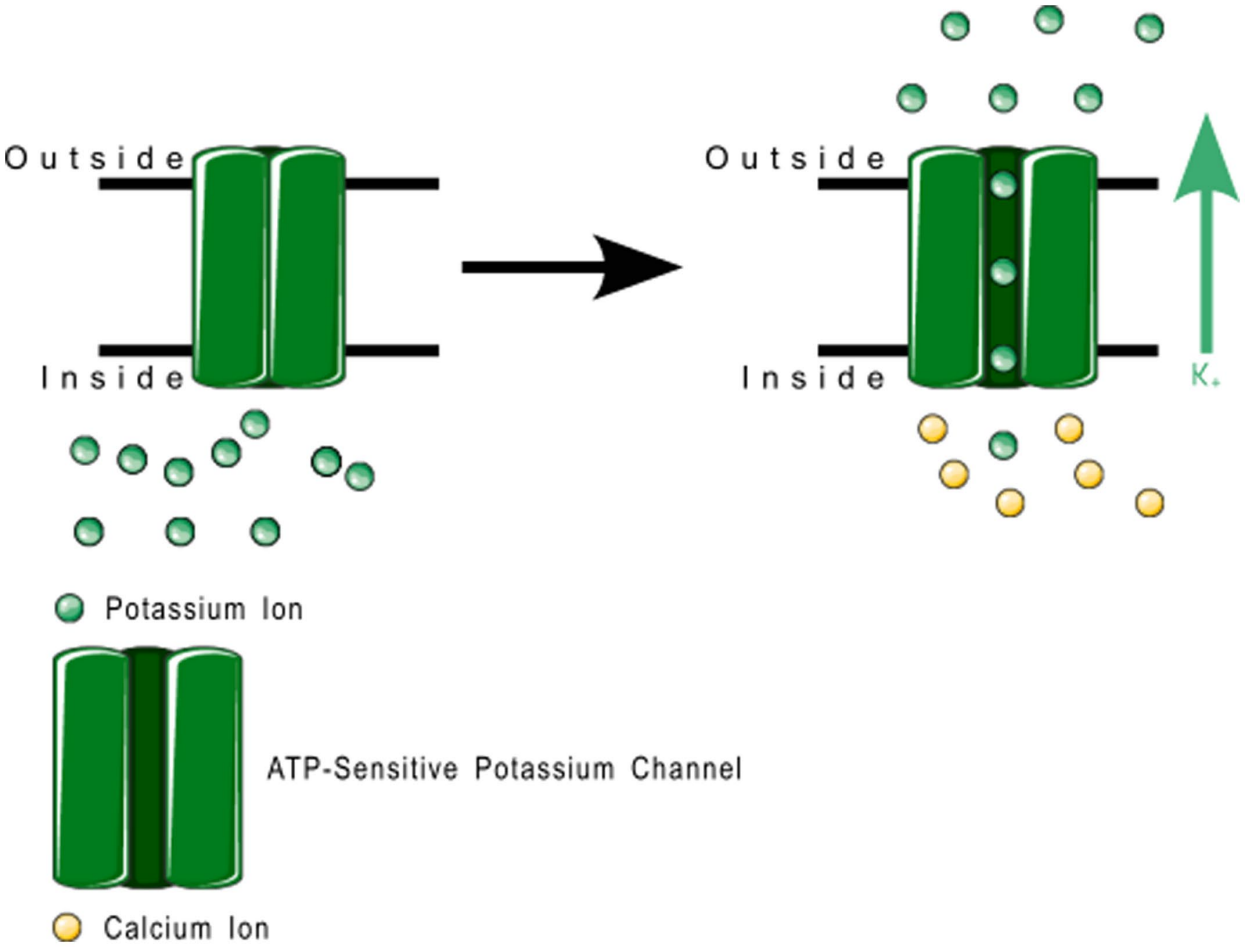
Small conductance calcium-gated potassium channel function. These channels regulate membrane depolarization by pushing potassium channels into the extracellular space.

**Figure 10:**
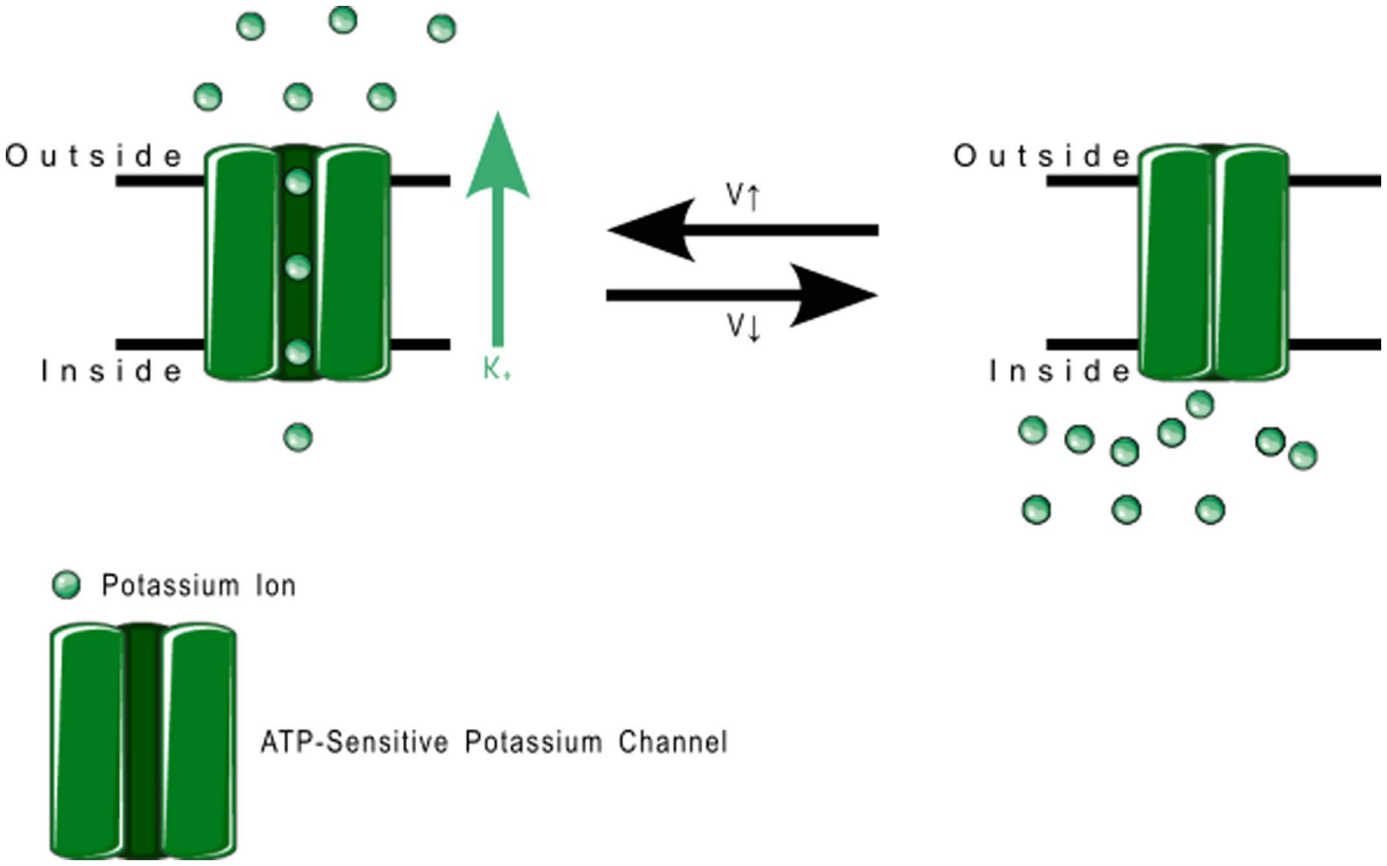
Voltage gated potassium channel family 7 function. KV7.2, 7.3, and 7.5 can heteromerize with each other to regulate the exact threshold voltage that opens the channel. These produce the slow-activating M current in the brain.

6TM channels are voltage-gated and calcium-gated potassium channels. The members of this family of channels are divided between voltage-gated channels (K_V_), which perform various functions, ranging from maintaining membrane potential in various organs to controlling the calcium signaling of leukocytes (85); and calcium-gated channels (K_Ca_), which are pleiotropic and mostly involved in the modulation of depolarization in the after hyperpolarization phase. The physiological function of K_Ca_4.x and K_Ca_5.x are not well understood (86). K_Ca_ channels are described in more detail in Table 3, and K_V_ in Table 4. Figure 8 shows the normal function of the large-conductance calcium-gated potassium channel (BK_Ca_). This channel regulates membrane potential by pushing potassium ions into the extracellular space. The potassium current induced by this channel results in a large efflux of potassium ions from the cytoplasm. Figure 9 shows the normal function of the small-conductance calcium-gated potassium channel (SK_Ca_). This channel acts in tandem with BK_Ca_ to regulate membrane potential by pushing potassium ions into the extracellular space. The potassium current induced by this channel results in a smaller efflux of ions than its ‘large’ counterpart. Figure 10 shows the normal function of the K_V_7.x channels; specifically, the channels formed by K_V_7.2, 7.3, and 7.5 channel proteins. The channels formed by these proteins are responsible for slow-activating outward currents (M currents) that moderate the membrane voltage by pushing potassium channels into the extracellular space to increase the threshold for triggering an action potential. These proteins can heteromerize with each other to form channels with a large range of threshold voltages.

**Table 3:** Calcium-gated potassium channels—data taken from several sources (39, 86, 181, 192, 193).

Subfamily	K_Ca_1.x	KCa2.x
Type	K_Ca_1.1	K_Ca_2.3
Function	Large-conductance Calcium-Activated and Voltage-Activated Mediation of after hyperpolarization	Small-conductance Calcium-Activated Mediation of Afterhyperpolarization
Activation	Ca^2+^, Voltage	Intracellular Ca^2+^
Inactivation	Voltage, controlled by β2- and β3-subunits	None
Activators	Intracellular Ca^2+^, NS1608, NS1619, BMS204352 (MaxiPost), DHS-1, estradiol, Mg^2+^	EBIO, riluzone, NS309
Inhibitors	None	None
Gating Inhibitors	None	None
Blockers	Triethanolamine, charybdotoxin, ineriotoxin, paxilline, slotoxin, Chinese scorpion toxin	leiurotoxin/scyllatoxin, apamin, Tskappa, Pi1-OH, Pi1-NH_2_, UCL1684, bicuculline, amitriptyline, fluoxetine, desipramine, imipramine, nortriptyline, fluphenazine, promethazine, chlorpromazine
Organ(s)	Ubiquitous	Brain, B lymphocytes, microglial cells, skeletal muscle, myometrium, prostate, kidney, heart, liver, pituitary gland, pancreas, colon, germinal cells, head, neck, ovary, vascular endothelium, Burkitt’s lymphoma
Channelopathies	Migraine, erectile dysfunction, incontinence, ataxia, auditory defects. Implicated in Alzheimer’s disease.	Implicated in Migraine, Alzheimer’s disease, age-related degeneration of hippocampus, schizophrenia, anorexia nervosa, spinocerebellar ataxia, hypertension, bladder instability. Protein levels and mRNA levels are increased in skeletal muscle after denervation or in myotonic muscular dystrophy

**Table 4:** Voltage-gated potassium channels—data taken from several sources (39, 85, 92, 181, 194–199).

Subfamily	K_V_7.x
Type	K_V_7.2
Function	Voltage-gated delayed rectifier channel that determines the subthreshold excitability of neurons, co-assembles with K_V_7.3 to form the slow-activating M current in brain, can replace K_V_7.3 in some presynaptic terminals
Activation	Membrane voltage above 26 mV
Inactivation	Membrane voltage below 18 mV
Activators	Retigabine, BMS204352 (MaxiPost)
Inhibitors	None
Gating Inhibitors	None
Blockers	Tetraethylammonium, XE991, linopiridine, L735821
Organ(s)	Brain, sympathetic ganglia, lungs, testis, heart, breast, eye, germ cells, placenta, small intestine, neuroblastomas
Channelopathies	Benign familial neonatal convulsions with myokymia; loss-of-function mutations result in increased sensitivity to seizure inducing chemicals; implicated in migraine
Note:	The M Current is critical in determining the subthreshold excitability of neurons and the responsiveness to synaptic inputs
Type	K_V_7.3
Function	Voltage-gated delayed rectifier channel that determines the subthreshold excitability of neurons, co-assembles with K_V_7.1 or K_V_7.5 to form the slow-activating M current in brain
Activation	Membrane voltage above 39 mV
Inactivation	Not established
Activators	Retigabine, ZXE991, BMS204352 (MaxiPost)
Inhibitors	None
Gating Inhibitors	None
Blockers	Tetraethylammonium, linopiridine
Organ(s)	Brain, testis, retina, colon, eye, head, neck
Channelopathies	Benign familial neonatal convulsions with myokymia; implicated in migraine, epilepsy and neurological impairment
Type	K_V_7.5
Function	Voltage-gated delayed rectifier current that determines the subthreshold excitability of neurons; co-assembles with K_V_7.3 to form the M current in brain
Activation	Membrane voltage above 30 mV
Inactivation	Not Established
Activators	Retigabine, BMS204352 (MaxiPost)
Inhibitors	None
Gating Inhibitors	None
Blockers	Tetraethylammonium, linopiridine, XE991
Organ(s)	Brain, sympathetic ganglia, skeletal muscle
Channelopathies	Implicated in epilepsy, migraine, and neurological impairment

**Figure 11:**
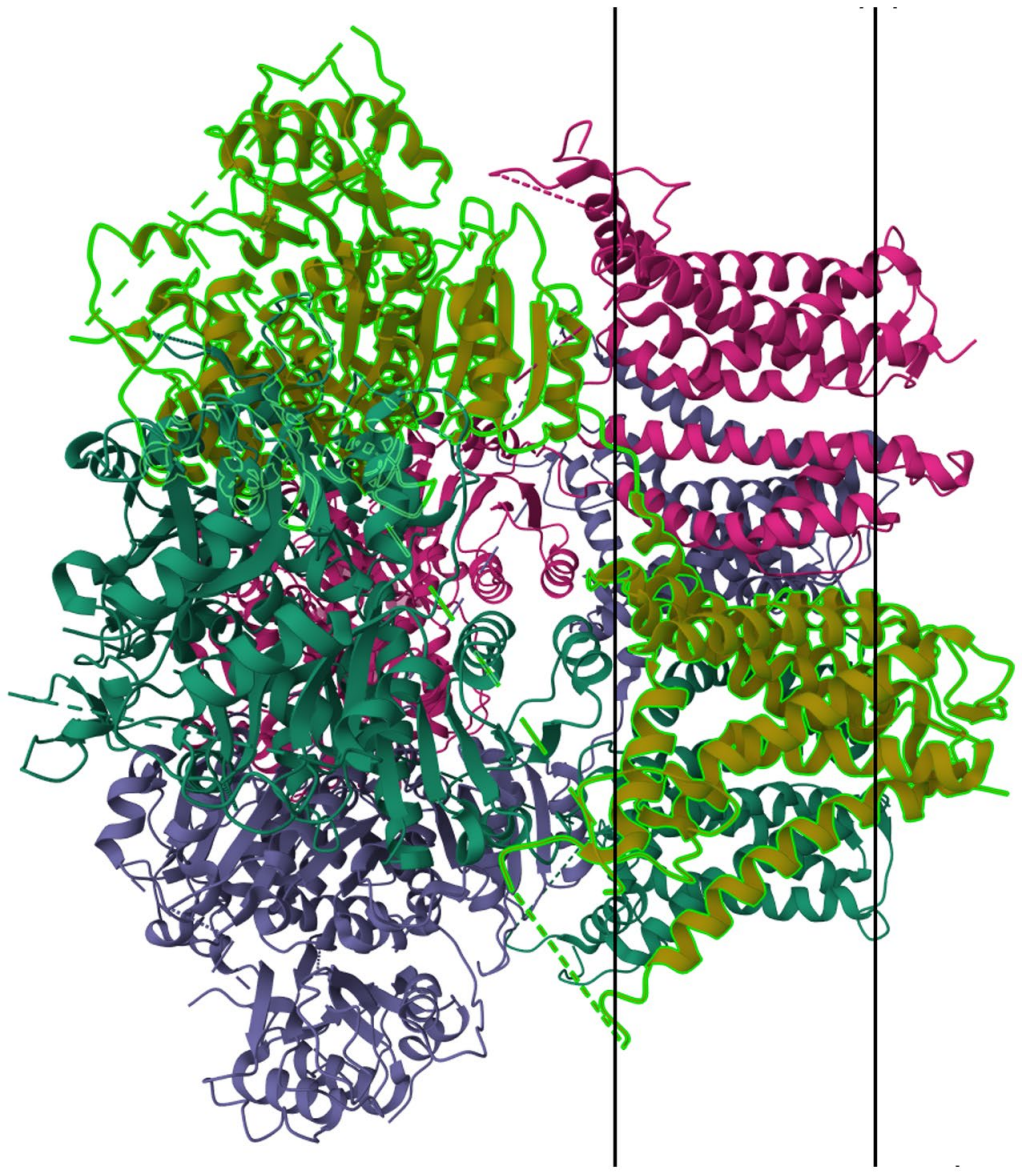
Protein model of BKCa channel (KCa1.1) tetramer. The molecule is oriented such that the cytosolic region falls to the left of the manuscript. The two surfaces of the cell membrane are denoted by two black bars. Image modified from Tao et al. (186) as retrieved from the RCSB Protein Data Bank.

**Figure 12:**
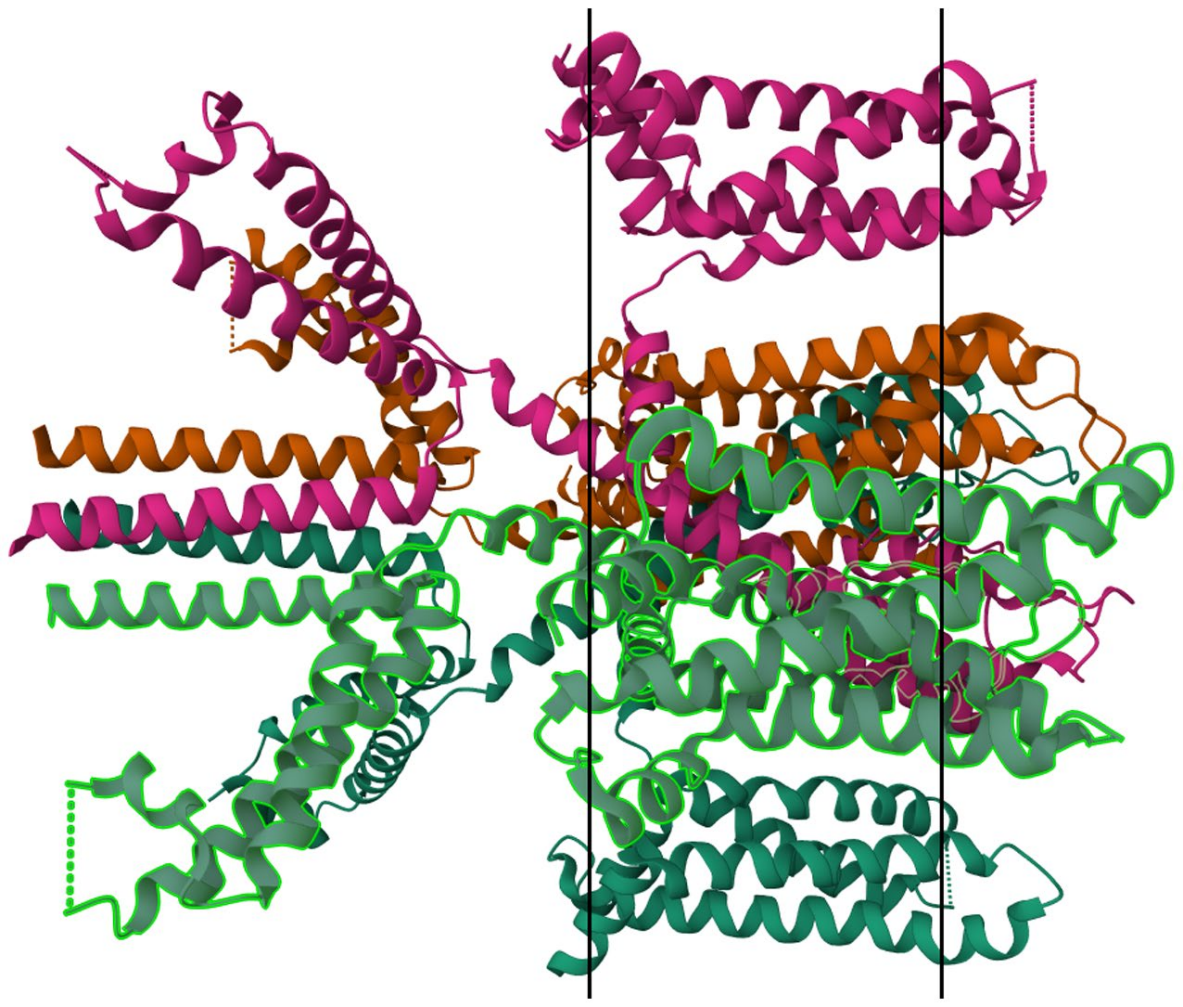
Protein model of KV7.1 tetramer. The molecule is oriented such that the cytosolic region falls to the left of the manuscript. The two surfaces of the cell membrane are denoted by two black bars. Image modified from Ma et al. (187) as retrieved from the RCSB Protein Data Bank.

A large number of articles have been published implicating voltage-gated and calcium-gated potassium channels in migraine pathogenesis, especially large-conductance calcium-gated potassium channels (BK_Ca_) and K_V_7. Episodic ataxias, characterized by sporadic bouts of severe discoordination, are closely related to familial hemiplegic migraine, and have been associated with voltage-gated potassium channels. Lerche, Mitrovic, and Lehmann-Horn in 1997 describe potassium channels as playing important roles in episodic ataxias (35). Ptácek in 1997 also mentions K_V_1.1 in association with episodic ataxias (87), as does Felix in 2000 (88) and Kullmann in 2002 (89). Lehmann-Horn and Jurkat-Rott in 1999 describe voltage-gated potassium channels K_V_1.1, 1.2 and 1.3 in convulsions and episodic ataxias (90). Gribkoff in 2003 already mentions the use of KV7 channel openers as analgesics, including retigabine, gabapentin, and flupirtine, and recommends further study on channel openers for migraine treatment (91). Gutman et al. — in their 2005 publication — discuss the role of K_V_7 in mediating the M current, a noninactivating current implicated in several neurological disorders including migraine and seizures (85). Wei et al. in their 2005 publication describe the effect of BK_Ca_ and Small-Conductance calcium-gated potassium channels (SK_Ca_) in epilepsy and headache (92). Maljevic et al. — separately in 2008 and 2010 — discuss the role of K_V_7 in disorders of neuronal hyperexcitability, implicating these channels in migraine and epilepsy among other disorders (93, 94). Maljevic and Lerche — in their 2013 paper — discuss the potential use of K_V_7 openers in treatment of neurological syndromes, but raise concerns as to side-effects due to the abundant distribution of K_V_ channels in various tissues (95). Citak et al. in 2022 describe the involvement of retigabine-induced K_V_7.x activation in reducing the CGRP release due to Transient Receptor Potential Ion Channel (TRP) activation, a phenomenon known in migraine (96). Several pharmaceutical compounds have also been patented or tested for their efficacy as migraine abortives based on their effect on voltage-gated or calcium-gated potassium channels (35, 85–91, 95–106). Figure 11 shows a ribbon model of the BKCa tetramer, and Figure 12 a ribbon model of the KV7.1 tetramer.

**Figure 13:**
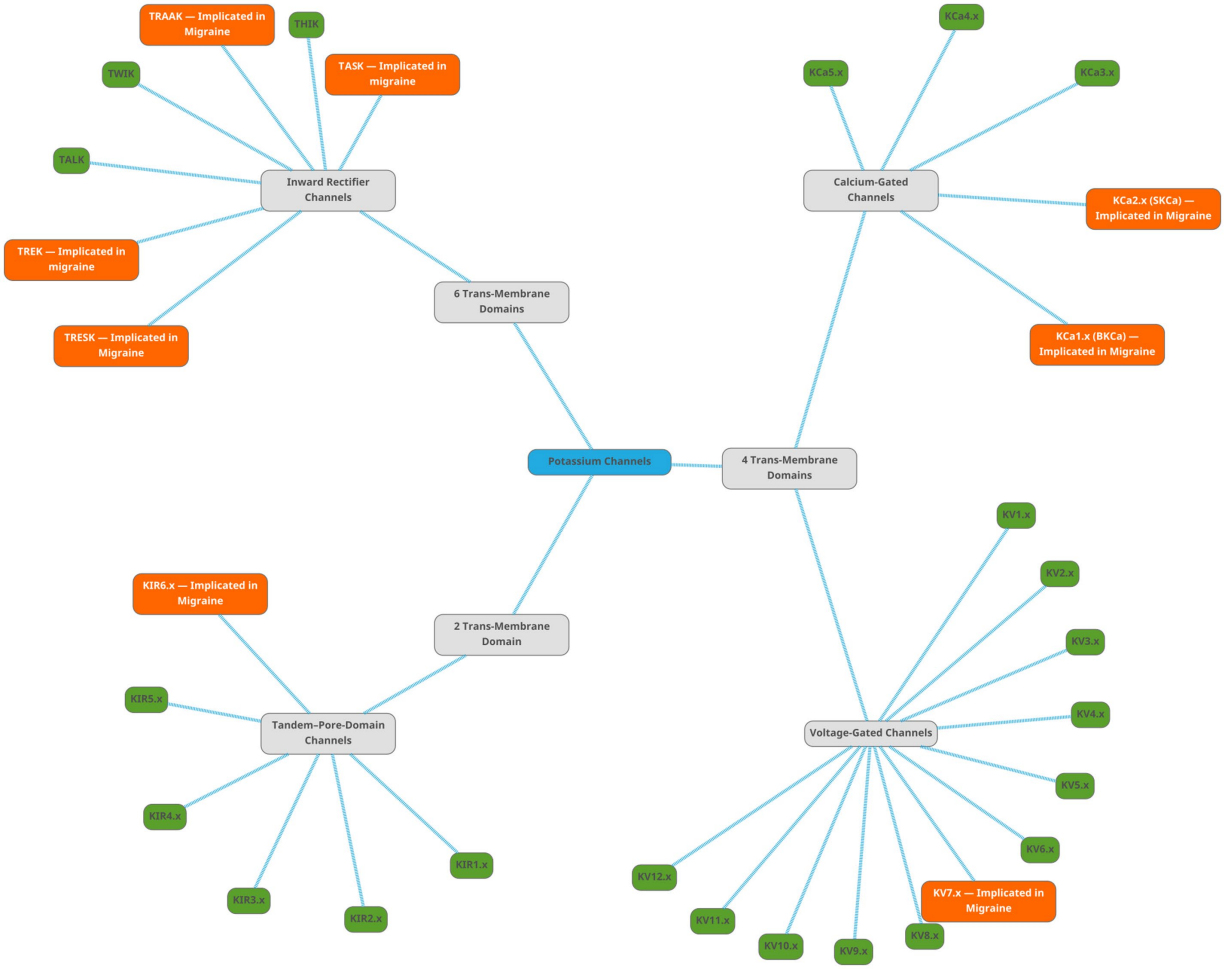
Mind map showing the subtypes of potassium channels. The root node, labelled ‘Potassium Channels’, is light-blue. There are three subtypes of potassium channels, shown as gray nodes. The families are shown as green nodes for families not implicated in migraine, and orange nodes for families implicated in migraine literature.

Figure 13 is a mind map of the subtypes of potassium channels. The figures representing the channel functions were created by using pictures from Servier Medical Art. Servier Medical Art by Servier is licensed under a Creative Commons Attribution 3.0 Unported License.^1^ The mind map was created using the free vector graphics editor Inkscape v. 1.3.2.^2^ The protein structure images were retrieved from the RSCB Protein Data Bank (107, 108) and the membrane markers added using the free image manipulation software GIMP 2.10.34.^3^

## 5 Discussion

### 5.1 The pathophysiology of migraine and the role of potassium channels

Migraine is a primary headache disorder that is caused by a spreading depolarization in cortical neurons (2, 5). Several inducing and exacerbating factors for cortical spreading depression are known and each individual case of migraine is likely to be a combination of several of these factors. A combination of genetic factors, environmental triggers, and physiological factors are implicated as affecting the intensity, frequency, and duration of migraine attacks, as well as the attendant symptoms and phenotypes (1, 5, 10, 15, 40, 109–114). The nociceptive symptoms of migraine are caused by signal induction in the trigeminal nerve system due to CSD waves perpetuating into TG afferents in the cortex. Ion channels and neurotransmitters in TG nerves and the nucleus caudalis are involved in the indigenous generation of pain impulses from polarization changes caused by the CSD (4, 16–18, 20–25, 27–30, 33, 34, 70, 72, 77, 78, 81, 83, 96, 102, 103, 115–121).

Cortical spreading depression has been known to induce extreme acute increase in extracellular potassium levels since 1974 (122–124). Other than the well-described role of ATP-Dependent Sodium-Potassium Pump genes in Familial Hemiplegic Migraine (10, 19, 40, 48, 52, 58, 89, 90, 110–114, 125–128), there have been several potassium channels implicated in migraine pathogenesis and progression in several papers over the last 20–25 years.

### 5.2 Classification of potassium channels

Potassium channels are classified into three families based on their molecular structure (39). 2 Trans-Membrane domain channels are generally responsible for rectifying neuronal polarization, 4TM channels are responsible for maintaining ion balance across the axonal membrane, and 6 TM channels are responsible for weak and strong modulatory currents in the CNS and in the heart and gut. Among the 2TM channels, K_ATP_ has been shown the highest promise as a target for migraine prophylaxis, especially in association with the SUR2_B_ modulatory subunit, with several drugs having been described and patented that that target the same. Among the 4TM channels, mutations in the TRESK protein that interact with TREK1 and 2 have been shown to be associated with certain pedigrees of migraine.

### 5.3 On the role played by potassium channels in migraine

#### 5.3.1 2TM channels

Downregulation of K_IR_ channels in astrocytes has been linked to altered nociception and one article (129) describes a potential link to migraine. When ATP-sensitive potassium channels were targeted for treatment of asthma and angina pectoris, K_ATP_ channel openers were found to induce migraine in migraine patients. While some papers have disputed this finding with opposing findings, the headaches reported as a side effect of levcromakalim and other K_ATP_ channel openers have been used as proof that K_ATP_ channels are involved in migraine pathogenesis (40, 68–75, 106, 130–132). Glibenclamide, a K_ATP_/SUR1A inhibitor has been tested extensively as a migraine abortive or suppressant due to its opposing effect on K_ATP_ to levcromakalim (68, 72, 133, 134), but results have so far been inconclusive. As glibenclamide targets SUR1A more than SUR1B, and as SUR1B mutations are more known in migraine pathogenesis, the latter has been suggested as a target for migraine prophylaxis (74).

#### 5.3.2 4TM channels

Two pore-domain open rectifier channels, especially TRESK (12, 40, 41, 43, 77, 79, 80, 83, 119, 120, 135–147), TREK-1 (84, 119, 144, 145, 148) and TASK-2 (144, 145) have been shown to be involved in cortical spreading depression and migraine nociception. Overexpression of TRESK in post-synaptic regions of neurons is linked to neurotransmitter dysmodulation (137); and is suspected to result in migraine due to serotonin or GABA dysfunction. Cloxyquin, a TRESK opener, has been studied as a migraine prophylactic (141, 149, 150). A particular frameshift mutation of TRESK has been described as closely segregating with migraine phenotype in certain pedigrees of migraine (10, 12, 41, 42, 78, 80, 114, 118, 138, 139, 141, 142, 144, 146, 151–167), and has been shown to have migraine-inducing effect due to its inhibitory effect on TREK-1 and TREK-2 proteins (81–84, 143, 168–170).

#### 5.3.3 6TM channels

Calcium-gated potassium channels such as large-conductance calcium-gated potassium channels (BK_Ca_ or MaxiK) are responsible for reducing inflammatory pain in both central and peripheral tissues (103, 104, 106, 171). Genes coding for small conductance calcium-gated potassium channels (SK_Ca_) such as KCNN3 have been associated with Familial Hemiplegic Migraine in some pedigrees (101), alongside the more well known CANCA1A and ATP1A2. BK_Ca_ channel openers such as MaxiPost have been shown clinically to induce migraine and other primary headache symptoms (104). Openers and blockers of potassium channels have been studied for treatment of neurological conditions including migraine with multiple papers published in the last 18–20 years (75, 91, 100, 172–174). Other targets for migraine treatment have been blockers of voltage-gated potassium channels such as benzylamide derivatives and other heterocyclic compounds and K_V_7 openers.

### 5.4 Therapeutic implications

Various potassium channels are currently known to play important roles in migraine pathogenesis. Targeting these potassium channels is very likely therefore to yield actionable results toward the treatment, prevention, abortion, or amelioration of migraine attacks and pain. However, potassium is a ubiquitous ion in the human body, involved in a vast array of disparate modulatory and sensory pathways in various organs in the body, including gastrointestinal, hepatic, cardiovascular, and lymphatic systems. Therefore, any treatment for migraine that targets potassium channel proteins would have to be capable of doing so in an organ-specific manner, so as to minimize extracranial side-effects, either by targeting accessory molecules, proteins, or peptides unique to potassium channels in neuronal tissue or by targeting specific functionality of potassium channels in neuronal tissue.

Pharmaceutical ingredients that act as K_V_7.x openers and partial inhibitors—including retigabine (91, 96, 97, 117, 175–177), ezogabine (178), flupirtine (91, 175, 177, 179), and linopiridine (91), along with several substituted acrylamide compounds (91, 97, 172, 173, 175–177, 180)—have been and are continuing to be tested as migraine abortives or prophylactics for nigh-on two decades. Loss of function mutations in voltage-gated potassium channels are linked to reduced sensitivity to analgesics including paracetamol and opioids (181). Tricyclic antidepressants such as amitriptyline act at least partially on voltage-gated potassium channels (83, 182), and this might be the target of action of tricyclic antidepressants as migraine prophylactics. Whereas several migraine prophylactic drugs are known to act at least in part through their effect on cerebral and vascular potassium channels no specific potassium channel modulator compounds have been released as commercial products yet. However, with modern techniques and computational analysis we can hope to find suitable candidates for targeted and specific anti-migraine prophylactics either from scratch or by repurposing compounds used in treatments for other channelopathies.

## 6 Summary and conclusion

Migraine, despite being one of the most common primary headache disorders, still has many unknowns in both the underlying mechanisms and in the treatment. The functional mechanism behind migraine is thought to be the triggering by environmental triggers of cortical spreading depression, which results in an adverse activation of nociceptive structures in the brain and CNS. Three neurochemicals are most closely associated with migraine: serotonin, CGRP, and *γ*-amino butyric acid (GABA) but several other pathways including Poly-Adenylate Cyclase Activating Peptide (PACAP) and Vasoactive Intestinal Peptide (VIP) are also involved. Migraine pathogenesis is also strongly associated with the functioning of neuronal ion channels, and familial hemiplegic migraine in particular has been linked to mutations in calcium channels, sodium channels, and sodium-potassium ATPase exchanger channels. However, these same channels do not seem to have any association to non-hemiplegic familial migraine with aura or migraine without aura. Non-hemiplegic familial migraine cases are still likely to be associated with some channel or other, and to date the most promising candidates have been potassium channels, with several articles (as discussed in detail above) associating these channels with migraine phenotypes in various regions.

Potassium channels are transmembrane proteins found in various tissues ranging from cardiac tissue and gastrointestinal tissue to neurons, and are involved in autocellular signaling and regulation of membrane potential. Potassium channels have been studied as potential actors in migraine pathogenesis for several years, and the current sum of understanding of migraine pathogenesis gives more than a little import to the proper functioning of potassium channels, especially those that are involved in membrane potential maintenance and in resetting neuronal potential after firing. Dysfunctions of potassium channels have been linked to several neurological disorders including long QT syndrome, epilepsy, Barrette’s syndrome, dementia and delirium, neurodevelopmental disorders, and migraine. Whereas gene mutations involving open rectifier channels such as TRESK, TREK, and TASK have been identified for a long time in familial non-hemiplegic migraine lines, current focus on treatment has been more focused on ATP-sensitive potassium channels and calcium- or voltage-dependent potassium channels. Several existing migraine prophylactics — including analgesics, antiepileptics, and antidepressants — have been shown to act against migraine at least partially via targeting one or more potassium channels. The targeting of potassium channels, either through activation or blocking of the channel function, is a constantly updating and novel research focus with primarily clinical and case–control or cohort studies. Identification of novel agents acting on potassium channels in the meningeal and cortical regions of the brain with minimal side-effects in other organs could lead to novel treatment of currently resistant migraine phenotypes.

Potassium channels in the cortex and in cerebral vasculature clearly play an important role in modulating both the neurological and vasodilatory symptoms of migraine. The current avenues of research on potassium channels are focused on clinical presentations and identification of channel function in migraine patients, but very little consideration has been given so far to approaches from the molecular analysis side. Future investigations should aim to elucidate both the exact function of potassium channels in migraine and the reasoning behind the observed effects of blocking or opening potassium channels in the cortex or the trigeminovascular system, and the discovery or repurposing of drugs and active pharmaceutical ingredients for the treatment of migraine via their interactions with potassium channels. The use of novel analytic and exploratory tools to better classify and diagnose migraine subtypes based on channel patterns is likely to result in a more designer-care angle to treatment of migraine, moving away from the current ‘migraine cocktail’ treatment to a more specialized treatment plan with less side effects and quicker action.

### 6.1 Future directions

Potassium channels are ubiquitous in the body and perform various roles, sometimes resulting in apparent conflicts where openers and closers of the same channel can appear to have similar effects, due to the two molecules being segregated to different parts of the body. It is still unknown whether small molecules that target potassium channel accessory proteins such as sulfonylurea receptors and regulatory domains could be used to treat migraine phenotypes. Current research is focused predominantly on describing the effect and functioning of potassium channels and has not made inroads into analysis and prediction of potential therapeutic agents or avenues of treatment. There exists therefore a striking gap in knowledge as to the pharmacological and therapeutic potential of the knowledge being discovered about the role of ion channels in primary neurogenic pain disorders including migraine. Investigations along these lines can most efficiently be done in a computational manner, integrating genomic, epigenomic, transcriptomic, and proteomic approaches to identify viable druggable targets and novel active pharmaceutical ingredients in a synergistic manner. Exploration into this field could result not only in novel therapeutic methods for migraine and other related headache disorders but also for anesthesia and analgesia in general. As the covert phenotype of migraine is thought to be related to epilepsy and other dyspolarization disorders, research into migraine therapeutics could also result in discovery of safer or more specific anticonvulsants and antiepileptic drugs as well.

## Footnotes

^1^ https://creativecommons.org/licenses/by/3.0/

^2^ https://inkscape.org/

^3^ https://gimp.org/

## Glossary

2TM: Two Transmembrane Domain Channel
4TM: Four Transmembrane Domain Channel
6TM: Six Transmembrane Domain Channel
ATP: Adenosine Tri-Phosphate
BK_Ca_: Large (Big) Conductance Calcium-Gated Potassium Channel
CGRP: Calcitonin Gene Related Peptide
CNS: Central Nervous System
CSD: Cortical Spreading Depression
GABA: Gamma Amino Butyric Acid
K_2P_: 2-Pore Domain Potassium Channel
K_ATP_: ATP-Sensitive Potassium Channel
K_Ca_: Calcium-Gated Potassium Channel
K_IR_: Inward Rectifying Potassium Channel
K_V_: Voltage-Gated Potassium Channel
PACAP: Poly Adenylate Cyclase Activating Peptide
RCSB: Research Collaboratory for Structural Bioinformatics
SK_Ca_: Small Conductance Calcium-Gated Potassium Channel
TASK: TWIK-Related Acid-Sensitive Potassium Channel
TRAAK: TWIK-Related Arachidonic Acid Sensitive Potassium Channel
TREK: TWIK Related Potassium Channel
TRESK: TWIK Related Spinal Cord Potassium Channel
TWIK: Tandem-Pore Domain Containing Weak Inward Rectifying Potassium Channel
VIP: Vasoactive Intestinal Peptide
