# Supplementary material for: Migraine is a dysfunction of neuronal potassium ion channels
Source: Front Neurol. 2025 Jul 31;16:1622994. doi: 10.3389/fneur.2025.1622994 (PMC12352164; doi:10.3389/fneur.2025.1622994)
Supplement: Supplementary file 1 [file Table_1.docx]

Table Relevant content from articles included based on their abstract

| **№** | **Reference Line** | **Relevant Content** | **Included** | |
| --- | --- | --- | --- | --- |
|  | McCoull D, Ococks E, Large JM, et al (2021) A “target class” screen to identify activators of two-pore domain potassium (K2P) channels. SLAS Discov 26:428–438. <https://doi.org/10.1177/2472555220976126> | None | | No |
|  | Pettingill P, Weir GA, Wei T, et al (2019) A causal role for TRESK loss of function in migraine mechanisms. Brain 142:3852–3867. <https://doi.org/10.1093/brain/awz342> | TRESK loss-of-function causes exaggerated migraine symptoms in migraine model; Cloxyquin prevents sensitization | | Yes |
|  | Nyholt DR, LaForge KS, Kallela M, et al (2008) A high-density association screen of 155 ion transport genes for involvement with common migraine. Human Molecular Genetics 17:3318–3331. <https://doi.org/10.1093/hmg/ddn227> | KCNB2 was implicated in combination with CACNB2 in Finnish FHM cohort | | Yes |
|  | Mössner R, Weichselbaum A, Marziniak M, et al (2005) A highly polymorphic poly-glutamine stretch in the potassium channel KCNN3 in migraine. Headache 45:132–136. <https://doi.org/10.1111/j.1526-4610.2005.05027.x> | KCNN3 mutation associated with migraine phenotype | | Yes |
|  | Staehr C, Rajanathan R, Postnov DD, et al (2020) Abnormal neurovascular coupling as a cause of excess cerebral vasodilation in familial migraine. Cardiovasc Res 116:2009–2020. <https://doi.org/10.1093/cvr/cvz306> | Increased expression of K_IR_2.1 is associated with hyperfusion in mouse model of migraine | | Yes |
|  | Raffaelli B, Do TP, Chaudhry BA, et al (2024) Activation of ATP-sensitive potassium channels triggers migraine attacks independent of calcitonin gene-related peptide receptors: a randomized placebo-controlled trial. Cephalalgia 44:03331024231222916. <https://doi.org/10.1177/03331024231222916> | Levcromakalim — K_ATP_ opener — induces migraine attack independent of CGRP block | | Yes |
|  | Lebedeva A, Plata A, Nosova O, et al (2018) Activity-dependent changes in transporter and potassium currents in hippocampal astrocytes. Brain Res Bull 136:37–43. <https://doi.org/10.1016/j.brainresbull.2017.08.015> | Increased extracellular K+ concentration induces depolarization | | Yes |
|  | Cady RJ, Shade CL, Cady RK (2012) Advances in drug development for acute migraine. Drugs 72:2187–2205. <https://doi.org/10.2165/11641120-000000000-00000> | None | | No |
|  | Sutherland HG, Albury CL, Griffiths LR (2019) Advances in genetics of migraine. The Journal of Headache and Pain 20:72. <https://doi.org/10.1186/s10194-019-1017-9> | TRESK loss-of-function is associated with migraine with aura | | Yes |
|  | Imbrici P, Nematian-Ardestani E, Hasan S, et al (2020) Altered functional properties of a missense variant in the TRESK K+ channel (TRESK) associated with migraine and intellectual disability. Pflugers Arch 472:923–930. <https://doi.org/10.1007/s00424-020-02382-5> | TRESK loss-of-function mutation associated with migraine with aura | | Yes |
|  | Harriott AM, Strother LC, Vila-Pueyo M, Holland PR (2019) Animal models of migraine and experimental techniques used to examine trigeminal sensory processing. The Journal of Headache and Pain 20:91. <https://doi.org/10.1186/s10194-019-1043-7> | Cerebral nociception is associated with decrease in calcium-dependent potassium current; triptans induce hyperpolarizing shifts in voltage-gated potassium currents | | Yes |
|  | Curtain R, Sundholm J, Lea R, et al (2005) Association analysis of a highly polymorphic CAG repeat in the human potassium channel gene KCNN3 and migraine susceptibility. BMC Med Genet 6:32. <https://doi.org/10.1186/1471-2350-6-32> | None | | No |
|  | Fernandez F, Curtain RP, Colson NJ, et al (2007) Association analysis of chromosome 1 migraine candidate genes. BMC Medical Genetics 8:57. <https://doi.org/10.1186/1471-2350-8-57> | None | | No |
|  | Christensen SL, Munro G, Petersen S, et al (2020) ATP sensitive potassium (KATP) channel inhibition: A promising new drug target for migraine. Cephalalgia 40:650–664. <https://doi.org/10.1177/0333102420925513> | K_ATP_ inhibitor glibenclamide attenuates cerebral hypersensitivity in mouse model of migraine | | Yes |
|  | Clement A, Guo S, Jansen-Olesen I, Christensen SL (2022) ATP-Sensitive potassium channels in migraine: Translational findings and therapeutic potential. Cells 11:2406. <https://doi.org/10.3390/cells11152406> | K_IR_6.1, K_ATP_, are involved in migraine triggering by CGRP, PACAP, NO etc | | Yes |
|  | Noseda R, Villanueva L (2023) Central generators of migraine and autonomic cephalalgias as targets for personalized pain management: Translational links. European Journal of Pain 27:1126–1138. <https://doi.org/10.1002/ejp.2158> | None | | No |
|  | Dieterich M (2007) Central vestibular disorders. Journal of Neurology 254:559–568. <https://doi.org/10.1007/s00415-006-0340-7> | None | | No |
|  | Kim J-B (2014) Channelopathies. Korean Journal of Pediatrics 57:1. <https://doi.org/10.3345/kjp.2014.57.1.1> | None | | No |
|  | Bernard G, Shevell MI (2008) Channelopathies: A review. Pediatric Neurology 38:73–85. <https://doi.org/10.1016/j.pediatrneurol.2007.09.007> | None | | No |
|  | Felix R (2000) Channelopathies: ion channel defects linked to heritable clinical disorders. J Med Genet 37:729–740. <https://doi.org/10.1136/jmg.37.10.729> | None | | No |
|  | Lengyel M, Erdélyi F, Pergel E, et al (2019) Chemically modified derivatives of the activator compound cloxyquin exert inhibitory effect on TRESK (K2P18.1) background potassium channel. Mol Pharmacol 95:652–660. <https://doi.org/10.1124/mol.118.115626> | None | | No |
|  | Mungoven TJ, Henderson LA, Meylakh N (2021) Chronic migraine pathophysiology and treatment: A review of current perspectives. Frontiers in Pain Research 2:705276. <https://doi.org/10.3389/fpain.2021.705276> | K_ATP_ openers induce headache | | No |
|  | Wright PD, Weir G, Cartland J, et al (2013) Cloxyquin (5-chloroquinolin-8-ol) is an activator of the two-pore domain potassium channel TRESK. Biochem Biophys Res Commun 441:463–468. <https://doi.org/10.1016/j.bbrc.2013.10.090> | None | | No |
|  | Jiang Z, Zhao L, Zhang X, et al (2021) Common variants in KCNK5 and FHL5 genes contributed to the susceptibility of migraine without aura in han chinese population. Sci Rep 11:6807. <https://doi.org/10.1038/s41598-021-86374-0> | KCNK5 mutation associated with increased susceptibility to migraine without aura | | Yes |
|  | Prachayasittikul V, Prachayasittikul S, Ruchirawat S, Prachayasittikul V (2018) Coriander (coriandrum sativum): A promising functional food toward the well-being. Food Research International 105:305–323. <https://doi.org/10.1016/j.foodres.2017.11.019> | None | | No |
|  | Kramer DR, Fujii T, Ohiorhenuan I, Liu CY (2016) Cortical spreading depolarization: Pathophysiology, implications, and future directions. J Clin Neurosci 24:22–27. <https://doi.org/10.1016/j.jocn.2015.08.004> | None | | No |
|  | Carneiro-Nascimento S, Levy D (2022) Cortical spreading depression and meningeal nociception. Neurobiol Pain 11:100091. <https://doi.org/10.1016/j.ynpai.2022.100091> | None | | No |
|  | Eikermann-Haerter K, Ayata C (2010) Cortical spreading depression and migraine. Curr Neurol Neurosci Rep 10:167–173. <https://doi.org/10.1007/s11910-010-0099-1> | None | | No |
|  | Ruppin E, Reggia JA (2001) Cortical spreading depression and the pathogenesis of brain disorders: a computational and neural network-based investigation. Neurol Res 23:447–456. <https://doi.org/10.1179/016164101101198839> | None | | No |
|  | Hershey AD (2010) Current approaches to the diagnosis and management of paediatric migraine. The Lancet Neurology 9:190–204. <https://doi.org/10.1016/S1474-4422(09)70303-5> | KCNN3 is associated with pediatric migraine | | Yes |
|  | Marmura MJ, Silberstein SD (2011) Current understanding and treatment of headache disorders: Five new things. Neurology 76:S31–S35. <https://doi.org/10.1212/WNL.0b013e31820c95cc> | TRESK frameshift mutation associated with migraine | | Yes |
|  | Goel K, Chhetri A, Ludhiadch A, Munshi A (2024) Current update on categorization of migraine subtypes on the basis of genetic variation: a systematic review. Molecular Neurobiology 61:4804–4833. <https://doi.org/10.1007/s12035-023-03837-3> | None | | No |
|  | Li K, McClenahan SJ, Han C, et al (2024) Discovery and characterization of VU0542270, the first selective inhibitor of vascular Kir6.1/SUR2B KATP ChannelsS. Mol Pharmacol 105:202–212. <https://doi.org/10.1124/molpharm.123.000783> | None | | No |
|  | Al-Karagholi MA-M, Ghanizada H, Kokoti L, et al (2020) Effect of KATP channel blocker glibenclamide on levcromakalim-induced headache. Cephalalgia 40:1045–1054. <https://doi.org/10.1177/0333102420949863> | K_ATP_ inhibitor delays headache onset from K_ATP_ openers | | Yes |
|  | Wang M, Urenjak J, Fedele E, Obrenovitch TP (2004) Effects of phosphodiesterase inhibition on cortical spreading depression and associated changes in extracellular cyclic GMP. Biochemical Pharmacology 67:1619–1627. <https://doi.org/10.1016/j.bcp.2003.12.029> | None | | No |
|  | Plane F, Garland CJ (1992) Electrophysiology of cerebral blood vessels. Pharmacol Ther 56:341–358. <https://doi.org/10.1016/0163-7258(92)90024-t> | None | | No |
|  | Menon S, Griffiths L (2013) Emerging genomic biomarkers in migraine. Future Neurology 8:87–101. <https://doi.org/10.2217/fnl.12.80> | KCNN3 and TRESK mutations are associated with migraine phenotype | | Yes |
|  | Tsantoulas C (2015) Emerging potassium channel targets for the treatment of pain. Curr Opin Support Palliat Care 9:147–154. <https://doi.org/10.1097/SPC.0000000000000131> | TRESK mutations are associated with migraine phenotype | | Yes |
|  | Wendt S, Wogram E, Korvers L, Kettenmann H (2016) Experimental cortical spreading depression induces NMDA receptor dependent potassium currents in microglia. J Neurosci 36:6165–6174. <https://doi.org/10.1523/JNEUROSCI.4498-15.2016> | CSD induces K_IR_ channel in microglia | | Yes |
|  | Ibrahim O, Sutherland HG, Maksemous N, et al (2020) Exploring neuronal vulnerability to head trauma using a whole exome approach. Journal of Neurotrauma 37:1870–1879. <https://doi.org/10.1089/neu.2019.6962> | None | | No |
|  | Al-Karagholi MA-M, Ghanizada H, Hansen JM, et al (2019) Extracranial activation of ATP-sensitive potassium channels induces vasodilation without nociceptive effects. Cephalalgia 39:1789–1797. <https://doi.org/10.1177/0333102419888490> | None | | No |
|  | Tolner EA, Houben T, Terwindt GM, et al (2015) From migraine genes to mechanisms. Pain 156:S64–S74. <https://doi.org/10.1097/01.j.pain.0000460346.00213.16> | TRESK is a modifier of migraine phenotype | | Yes |
|  | Liu P, Xiao Z, Ren F, et al (2013) Functional analysis of a migraine-associated TRESK K+ channel mutation. J Neurosci 33:12810–12824. <https://doi.org/10.1523/JNEUROSCI.1237-13.2013> | Loss-of-Function TRESK mutations result in lowered threshold for CSD | | Yes |
|  | Andres-Enguix I, Shang L, Stansfeld PJ, et al (2012) Functional analysis of missense variants in the TRESK (TRESK) K + channel. Scientific Reports 2:237. <https://doi.org/10.1038/srep00237> | TRESK mutation is not sufficient to cause migraine in the absence of other factors | | Yes |
|  | Gadgaard C, Jensen AA (2020) Functional characterization of 5-HT1A and 5-HT1B serotonin receptor signaling through g-protein-activated inwardly rectifying K+ channels in a fluorescence-based membrane potential assay. Biochem Pharmacol 175:113870. <https://doi.org/10.1016/j.bcp.2020.113870> | GIRK2 channel is involved in serotonin receptor role in migraine | | Yes |
|  | De Vries B, Haan J, Frants RR, et al (2006) Genetic biomarkers for migraine. Headache 46:1059–1068. <https://doi.org/10.1111/j.1526-4610.2006.00499.x> | None | | No |
|  | Gosalia H, Karsan N, Goadsby PJ (2023) Genetic mechanisms of migraine: Insights from monogenic migraine mutations. International Journal of Molecular Sciences 24:12697. <https://doi.org/10.3390/ijms241612697> | TRESK frameshift mutation is associated with familial migraine with aura | | Yes |
|  | Hanna MG (2006) Genetic neurological channelopathies. Nature Clinical Practice Neurology 2:252–263. <https://doi.org/10.1038/ncpneuro0178> | None | | No |
|  | Zorina-Lichtenwalter K, Meloto CB, Khoury S, Diatchenko L (2016) Genetic predictors of human chronic pain conditions. Neuroscience 338:36–62. <https://doi.org/10.1016/j.neuroscience.2016.04.041> | TRESK, KCNG4, KCNAB3, KCNN3 are associated with migraine phenotype | | Yes |
|  | Hershey AD (2008) Genetics of headache in children: where are we headed? Curr Pain Headache Rep 12:367–372. <https://doi.org/10.1007/s11916-008-0062-x> | Mutations resulting in longer polyglutamine tail of KCNN3 is associated with FHM | | Yes |
|  | Haan J, Terwindt GM, Ferrari MD (1997) Genetics of migraine. Neurologic Clinics 15:43–60. <https://doi.org/10.1016/S0733-8619(05)70294-2> | None | | No |
|  | De Boer I, Terwindt GM, Van Den Maagdenberg AMJM (2020) Genetics of migraine aura: An update. The Journal of Headache and Pain 21:64. <https://doi.org/10.1186/s10194-020-01125-2> | TRESK is associated with familial migraine with aura | | Yes |
|  | Hershey AD (2007) Genetics of migraine headache in children. Current Pain and Headache Reports 11:390–395. <https://doi.org/10.1007/s11916-007-0222-4> | KCNN3 is associated with migraine phenotype | | Yes |
|  | Gardner KL (2006) Genetics of migraine: an update. Headache 46:S19-24. <https://doi.org/10.1111/j.1526-4610.2006.00486.x> | None | | No |
|  | Sutherland HG, Jenkins B, Griffiths LR (2024) Genetics of migraine: complexity, implications, and potential clinical applications. The Lancet Neurology 23:429–446. <https://doi.org/10.1016/S1474-4422(24)00026-7> | None | | No |
|  | Sutherland HG, Griffiths LR (2017) Genetics of migraine: Insights into the molecular basis of migraine disorders. Headache: The Journal of Head and Face Pain 57:537–569. <https://doi.org/10.1111/head.13053> | TRESK mutation associated with migraine | | Yes |
|  | Grangeon L, Lange KS, Waliszewska-Prosół M, et al (2023) Genetics of migraine: where are we now? The Journal of Headache and Pain 24:12. <https://doi.org/10.1186/s10194-023-01547-8> | None | | No |
|  | Kokoti L, Al-Karagholi MA-M, Waldorff Nielsen CA, Ashina M (2023) Glibenclamide posttreatment does not inhibit levcromakalim induced headache in healthy participants: A randomized clinical trial. Neurotherapeutics 20:389–398. <https://doi.org/10.1007/s13311-023-01350-y> | None | | No |
|  | Ali MD, Gayasuddin Qur F, Alam MdS, et al (2023) Global epidemiology, clinical features, diagnosis and current therapeutic novelties in migraine therapy and their prevention: A narrative review. Curr Pharm Des 29:3295–3311. <https://doi.org/10.2174/0113816128266227231205114320> | TRESK channel protein frameshift mutation is associated with migraine | | Yes |
|  | Goadsby P (1997) How do the currently used prophylactic agents work in migraine? Cephalalgia 17:85–92. <https://doi.org/10.1046/j.1468-2982.1997.1702085.x> | None | | No |
|  | Cutrer FM, Smith JH (2013) Human studies in the pathophysiology of migraine: Genetics and functional neuroimaging. Headache: The Journal of Head and Face Pain 53:401–412. <https://doi.org/10.1111/head.12024> | TRESK and KCNN3 mutations have been associated with migraine pedigrees | | Yes |
|  | Al-Khazali HM, Christensen RH, Dodick DW, et al (2023) Hypersensitivity to opening of ATP-sensitive potassium channels in post-traumatic headache. Cephalalgia 43:03331024231210930. <https://doi.org/10.1177/03331024231210930> | None | | No |
|  | Maher BH, Griffiths LR (2011) Identification of molecular genetic factors that influence migraine. Molecular Genetics and Genomics 285:433–446. <https://doi.org/10.1007/s00438-011-0622-3> | TRESK mutation is associated with migraine with aura pedigree | | Yes |
|  | Haberberger RV, Barry C, Matusica D (2020) Immortalized dorsal root ganglion neuron cell lines. Frontiers in Cellular Neuroscience 14:184. <https://doi.org/10.3389/fncel.2020.00184> | None | | No |
|  | Al-Khazali HM, Deligianni CI, Pellesi L, et al (2024) Induction of cluster headache after opening of adenosine triphosphate-sensitive potassium channels: a randomized clinical trial. Pain 165:1289–1303. <https://doi.org/10.1097/j.pain.0000000000003130> | None | | No |
|  | Akerman S, Holland PR, Lasalandra MP, Goadsby PJ (2010) Inhibition of trigeminovascular dural nociceptive afferents by Ca(2+)-activated K(+) (MaxiK/BK(Ca)) channel opening. Pain 151:128–136. <https://doi.org/10.1016/j.pain.2010.06.028> | MaxiK openers can inhibit trigeminovascular nociception — the major cause of migraine pain | | Yes |
|  | Al-Karagholi MA-M (2023) Involvement of potassium channel signalling in migraine pathophysiology. Pharmaceuticals (Basel) 16:438. <https://doi.org/10.3390/ph16030438> | K_ATP_ and BKCa channels are involved in CGRP induced migraine phenotype | | Yes |
|  | Eren-Koçak E, Dalkara T (2021) Ion channel dysfunction and neuroinflammation in migraine and depression. Frontiers in Pharmacology 12:777607. <https://doi.org/10.3389/fphar.2021.777607> | KCNK5 and TRESK are involved in regulation of trigeminal nociception | | Yes |
|  | Albury CL, Stuart S, Haupt LM, Griffiths LR (2017) Ion channelopathies and migraine pathogenesis. Mol Genet Genomics 292:729–739. <https://doi.org/10.1007/s00438-017-1317-1> | TRESK is involved in regulation of trigeminal nociception and mutations are associated with migraine pedigrees | | Yes |
|  | Yan J, Dussor G (2014) Ion channels and migraine. Headache 54:619–639. <https://doi.org/10.1111/head.12323> | TRESK and BKCa are involved in trigeminovascular nociception | | Yes |
|  | Ploug K, Amrutkar D, Baun M, et al (2012) K(ATP) channel openers in the trigeminovascular system. Cephalalgia 32:55–65. <https://doi.org/10.1177/0333102411430266> | Migraine inducing effect of K_ATP_ openers is likely due to interaction with SUR2B | | Yes |
|  | Rainero I, Rubino E, Gallone S, et al (2014) KCNK18 (TRESK) genetic variants in italian patients with migraine. Headache 54:1515–1522. <https://doi.org/10.1111/head.12439> | Change of function TRESK mutations are prevalent in migraine patients | | Yes |
|  | Aiba I, Noebels JL (2021) Kcnq2/Kv7.2 controls the threshold and bi-hemispheric symmetry of cortical spreading depolarization. Brain 144:2863–2878. <https://doi.org/10.1093/brain/awab141> | K_V_7.2 controls CSD threshold, potential therapeutic target for CSD | | Yes |
|  | Kokoti L, Al-Karagholi MA-M, Ashina M (2020) Latest insights into the pathophysiology of migraine: the ATP-Sensitive potassium channels. Curr Pain Headache Rep 24:77. <https://doi.org/10.1007/s11916-020-00911-6> | K_ATP_ is involved in cyclic nucleotide phosphate cascade cause of migraine | | Yes |
|  | Alpay B, Cimen B, Akaydin E, et al (2023) Levcromakalim provokes an acute rapid-onset migraine-like phenotype without inducing cortical spreading depolarization. The Journal of Headache and Pain 24:93. <https://doi.org/10.1186/s10194-023-01627-9> | K_ATP_ opener levcromakalim provokes migraine like phenotype without inducing CSD | | Yes |
|  | Al‐Karagholi MA, Ghanizada H, Hansen JM, et al (2019) Levcromakalim, an adenosine triphosphate-sensitive potassium channel opener, dilates extracerebral but not cerebral arteries. Headache 59:1468–1480. <https://doi.org/10.1111/head.13634> | None | | No |
|  | Della Pietra A, Gómez Dabó L, Mikulenka P, et al (2024) Mechanosensitive receptors in migraine: a systematic review. The Journal of Headache and Pain 25:6. <https://doi.org/10.1186/s10194-023-01710-1> | TREK1 and TREK2 inhibition increases neuronal excitability and activation of the same suppresses migraine pain symptoms; TRESK frameshift mutation is linked to TREK-inhibitory protein | | Yes |
|  | Verkest C, Häfner S, Ávalos Prado P, et al (2021) Migraine and two-pore-domain potassium channels. Neuroscientist 27:268–284. <https://doi.org/10.1177/1073858420940949> | TRESK, TREK1 and TREK2 play a role in neuronal excitability, and are potential targets for treatment of migraine pain | | Yes |
|  | Silberstein SD, Dodick DW (2013) Migraine genetics: Part II. Headache: The Journal of Head and Face Pain 53:1218–1229. <https://doi.org/10.1111/head.12169> | TRESK frameshift mutation has been seen associated with migraine phenotypes | | Yes |
|  | Tajti J, Párdutz Á, Vámos E, et al (2011) Migraine is a neuronal disease. Journal of Neural Transmission 118:511–524. <https://doi.org/10.1007/s00702-010-0515-3> | None | | No |
|  | Rainero I, Roveta F, Vacca A, et al (2020) Migraine pathways and the identification of novel therapeutic targets. Expert Opinion on Therapeutic Targets 24:245–253. <https://doi.org/10.1080/14728222.2020.1728255> | TRESK frameshift mutation has been seen associated with migraine phenotypes F139WfsX24 | | Yes |
|  | Juan Y, Shu O, Jinhe L, et al (2017) Migraine prevention with percutaneous mastoid electrical stimulator: A randomized double-blind controlled trial. Cephalalgia 37:1248–1256. <https://doi.org/10.1177/0333102416678623> | None | | No |
|  | Silvestro M, Iannone LF, Orologio I, et al (2023) Migraine treatment: Towards new pharmacological targets. Int J Mol Sci 24:12268. <https://doi.org/10.3390/ijms241512268> | K_ATP_ and BKCa channel openers cause migraine-like phenotype in migraineurs | | Yes |
|  | Royal P, Andres-Bilbe A, Ávalos Prado P, et al (2019) Migraine-associated TRESK mutations increase neuronal excitability through alternative translation initiation and inhibition of TREK. Neuron 101:232-245.e6. <https://doi.org/10.1016/j.neuron.2018.11.039> | TRESK mutation TRESK-MT associated with migraine results in the formation of secondary peptides that block TREK1 and TREK2 | | Yes |
|  | Rainero I, Vacca A, Govone F, et al (2019) Migraine: Genetic variants and clinical phenotypes. Curr Med Chem 26:6207–6221. <https://doi.org/10.2174/0929867325666180719120215> | None | | No |
|  | Lafrenière RG, Rouleau GA (2011) Migraine: Role of the TRESK two-pore potassium channel. Int J Biochem Cell Biol 43:1533–1536. <https://doi.org/10.1016/j.biocel.2011.08.002> | TRESK inhibition and loss-of-function can induce or exacerbate migraine phenotype | | Yes |
|  | Huntemann N, Bittner S, Bock S, et al (2022) Mini-review: Two brothers in crime - the interplay of TRESK and TREK in human diseases. Neurosci Lett 769:136376. <https://doi.org/10.1016/j.neulet.2021.136376> | TRESK mutations associated with migraine result in the formation of secondary peptides that block TREK1 and TREK2 | | Yes |
|  | Suleimanova A, Talanov M, Gafurov O, et al (2020) Modeling a nociceptive neuro-immune synapse activated by ATP and 5-HT in meninges: Novel clues on transduction of chemical signals into persistent or rhythmic neuronal firing. Front Cell Neurosci 14:135. <https://doi.org/10.3389/fncel.2020.00135> | None | | No |
|  | Ayajiki K, Fujioka H, Noda K, et al (2001) Modifications by sumatriptan and acetylcholine of nitric oxide-mediated neurogenic dilatation in dog cerebral arteries. Eur J Pharmacol 420:67–72. <https://doi.org/10.1016/s0014-2999(01)01019-6> | None | | No |
|  | Callejo G, Giblin JP, Gasull X (2013) Modulation of TRESK background K+ channel by membrane stretch. PLoS One 8:e64471. <https://doi.org/10.1371/journal.pone.0064471> | None | | No |
|  | Villatoro-Gómez K, Pacheco-Rojas DO, Moreno-Galindo EG, et al (2018) Molecular determinants of Kv7.1/KCNE1 channel inhibition by amitriptyline. Biochem Pharmacol 152:264–271. <https://doi.org/10.1016/j.bcp.2018.03.016> | None | | No |
|  | Kowalska M, Prendecki M, Kozubski W, et al (2016) Molecular factors in migraine. Oncotarget 7:50708–50718. <https://doi.org/10.18632/oncotarget.9367> | TRESK frameshift mutation has been seen associated with migraine phenotypes | | Yes |
|  | The International Headache Genetics Consortium, Yang Y, Zhao H, et al (2018) Molecular genetic overlap between migraine and major depressive disorder. Eur J Hum Genet 26:1202–1216. <https://doi.org/10.1038/s41431-018-0150-2> | None | | No |
|  | Wulf H, Hay-Schmidt A, Poulsen AN, et al (2008) Molecular studies of BKCa channels in intracranial arteries: presence and localization. Cell Tissue Res 334:359–369. <https://doi.org/10.1007/s00441-008-0701-x> | None | | No |
|  | Maljevic S, Wuttke TV, Lerche H (2008) Nervous system KV7 disorders: breakdown of a subthreshold brake. J Physiol 586:1791–1801. <https://doi.org/10.1113/jphysiol.2008.150656> | None | | No |
|  | Wells E, Hacohen Y, Waldman A, et al (2018) Neuroimmune disorders of the central nervous system in children in the molecular era. Nature Reviews Neurology 14:433–445. <https://doi.org/10.1038/s41582-018-0024-9> | None | | No |
|  | Kullmann DM (2010) Neurological channelopathies. Annual Review of Neuroscience 33:151–172. <https://doi.org/10.1146/annurev-neuro-060909-153122> | None | | No |
|  | Davies NP, Hanna MG (1999) Neurological channelopathies: Diagnosis and therapy in the new millennium. Annals of Medicine 31:406–420. <https://doi.org/10.3109/07853899908998798> | None | | No |
|  | Kullmann DM, Hanna MG (2002) Neurological disorders caused by inherited ion-channel mutations. The Lancet Neurology 1:157–166. <https://doi.org/10.1016/S1474-4422(02)00071-6> | None | | No |
|  | Guo Z, Liu P, Ren F, Cao Y-Q (2014) Nonmigraine-associated TRESK K+ channel variant C110R does not increase the excitability of trigeminal ganglion neurons. J Neurophysiol 112:568–579. <https://doi.org/10.1152/jn.00267.2014> | Frameshift TRESK mutation associated with migraine increases hyperexcitability of nerves in trigeminal ganglion. | | Yes |
|  | Chen S, Ayata C (2017) Novel therapeutic targets against spreading depression. Headache: The Journal of Head and Face Pain 57:1340–1358. <https://doi.org/10.1111/head.13154> | TRESK frameshift mutation segregates perfectly with migraine with aura | | Yes |
|  | Nisar A, Ahmed Z, Yuan H (2023) Novel therapeutic targets for migraine. Biomedicines 11:569. <https://doi.org/10.3390/biomedicines11020569> | SUR2_B_ blockers are potential migraine treatments | | Yes |
|  | Al-Karagholi MA-M, Ghanizada H, Nielsen CAW, et al (2021) Opening of ATP sensitive potassium channels causes migraine attacks with aura. Brain 144:2322–2332. <https://doi.org/10.1093/brain/awab136> | K_ATP_ openers can trigger migraine aura and headache | | Yes |
|  | Al-Karagholi MA-M, Hansen JM, Guo S, et al (2019) Opening of ATP-sensitive potassium channels causes migraine attacks: a new target for the treatment of migraine. Brain 142:2644–2654. <https://doi.org/10.1093/brain/awz199> | K_ATP_ blockers are recommended as potential antimigraine drugs | | Yes |
|  | Al-Karagholi MA-M, Ghanizada H, Nielsen CAW, et al (2020) Opening of BKCa channels alters cerebral hemodynamic and causes headache in healthy volunteers. Cephalalgia 40:1145–1154. <https://doi.org/10.1177/0333102420940681> | BKCa openers induce headaches in healthy volunteers | | Yes |
|  | Al-Karagholi MA-M, Ghanizada H, Waldorff Nielsen CA, et al (2021) Opening of BKCa channels causes migraine attacks: a new downstream target for the treatment of migraine. Pain 162:2512–2520. <https://doi.org/10.1097/j.pain.0000000000002238> | BKCa blockers are recommended as potential antimigraine drugs | | Yes |
|  | Guo Z, Cao Y-Q (2014) Over-expression of TRESK K(+) channels reduces the excitability of trigeminal ganglion nociceptors. PLoS One 9:e87029. <https://doi.org/10.1371/journal.pone.0087029> | TRESK overexpression attenuates trigeminal nociception; TRESK openers are recommended as therapeutic agents for amelioration of migraine pain | | Yes |
|  | Cregg R, Momin A, Rugiero F, et al (2010) Pain channelopathies. J Physiol 588:1897–1904. <https://doi.org/10.1113/jphysiol.2010.187807> | None | | No |
|  | Davies AJ, Kim YH, Oh SB (2010) Painful neuron-microglia interactions in the trigeminal sensory system. The Open Pain Journal 3:14–28. <https://doi.org/10.2174/1876386301003010014> | None | | No |
|  | Haanes KA, Edvinsson L (2019) Pathophysiological mechanisms in migraine and the identification of new therapeutic targets. CNS Drugs 33:525–537. <https://doi.org/10.1007/s40263-019-00630-6> | hyperpolarization-activated cyclic nucleotide-gated channels are activated by K_ATP_ opening; which is interpreted as nociception in migraine | | Yes |
|  | Sehgal A, Hassan M, Rashid S (2014) Pharmacoinformatics elucidation of potential drug targets against migraine to target ion channel protein KCNK18. Drug Des Devel Ther 8:571. <https://doi.org/10.2147/DDDT.S63096> | None | | No |
|  | Dyhring T, Jansen-Olesen I, Christophersen P, Olesen J (2023) Pharmacological profiling of KATP channel modulators: An outlook for new treatment opportunities for migraine. Pharmaceuticals (Basel) 16:225. <https://doi.org/10.3390/ph16020225> | None | | No |
|  | Bulman D (1997) Phenotype variation and newcomers in ion channel disorders. Human Molecular Genetics 6:1679–1685. <https://doi.org/10.1093/hmg/6.10.1679> | None | | No |
|  | Rahm A, Gierten J, Kisselbach J, et al (2012) PKC-dependent activation of human K(2P) 18.1 K(+) channels. Br J Pharmacol 166:764–773. <https://doi.org/10.1111/j.1476-5381.2011.01813.x> | None | | No |
|  | Du X, Gamper N (2013) Potassium channels in peripheral pain pathways: expression, function and therapeutic potential. Curr Neuropharmacol 11:621–640. <https://doi.org/10.2174/1570159X113119990042> | K_V_7 channel openers show efficacy in treatment of migraine pain | | Yes |
|  | Maljevic S, Lerche H (2013) Potassium channels: a review of broadening therapeutic possibilities for neurological diseases. J Neurol 260:2201–2211. <https://doi.org/10.1007/s00415-012-6727-8> | K_V_7 channel openers have been suggested for treatment of migraine pain | | Yes |
|  | Enyedi P, Czirják G (2015) Properties, regulation, pharmacology, and functions of the k₂p channel, TRESK. Pflugers Arch 467:945–958. <https://doi.org/10.1007/s00424-014-1634-8> | TRESK frameshift mutation is associated with migraineurs in a migraine pedigree | | Yes |
|  | Pope L, Arrigoni C, Lou H, et al (2018) Protein and chemical determinants of BL-1249 action and selectivity for K2P channels. ACS Chem Neurosci 9:3153–3165. <https://doi.org/10.1021/acschemneuro.8b00337> | None | | No |
|  | Bjornsdottir G, Chalmer MA, Stefansdottir L, et al (2023) Rare variants with large effects provide functional insights into the pathology of migraine subtypes, with and without aura. Nature Genetics 55:1843–1853. <https://doi.org/10.1038/s41588-023-01538-0> | KCNK5 mutation showed protective effect against migraine attacks | | Yes |
|  | Wu Y-J, Dworetzky S (2005) Recent developments on KCNQ potassium channel openers. Curr Med Chem 12:453–460. <https://doi.org/10.2174/0929867053363045> | None | | No |
|  | Ávalos Prado P, Chassot A-A, Landra-Willm A, Sandoz G (2022) Regulation of two-pore-domain potassium TREK channels and their involvement in pain perception and migraine. Neurosci Lett 773:136494. <https://doi.org/10.1016/j.neulet.2022.136494> | TRESK frameshift mutation associated with migraine results in the transcription of an inhibitory molecule against TREK1 and TREK2, increasing the hyperexcitability of trigeminal neurons. | | Yes |
|  | LaPaglia DM, Sapio MR, Burbelo PD, et al (2018) RNA-Seq investigations of human post-mortem trigeminal ganglia. Cephalalgia 38:912–932. <https://doi.org/10.1177/0333102417720216> | None | | No |
|  | Lengyel M, Dobolyi A, Czirják G, Enyedi P (2017) Selective and state-dependent activation of TRESK (K2P 18.1) background potassium channel by cloxyquin. Br J Pharmacol 174:2102–2113. <https://doi.org/10.1111/bph.13821> | None | | No |
|  | Cader ZM (2003) Significant linkage to migrane with aura on chromosome 11q24. Human Molecular Genetics 12:2511–2517. <https://doi.org/10.1093/hmg/ddg252> | None | | No |
|  | Natale AM, Deal PE, Minor DL (2021) Structural insights into the mechanisms and pharmacology of K2P potassium channels. J Mol Biol 433:166995. <https://doi.org/10.1016/j.jmb.2021.166995> | None | | No |
|  | Wu Y-J, He H, Sun L-Q, et al (2004) Synthesis and structure-activity relationship of acrylamides as KCNQ2 potassium channel openers. J Med Chem 47:2887–2896. <https://doi.org/10.1021/jm0305826> | KCNQ2 openers demonstrated significant activity in migraine models | | Yes |
|  | Al-Karagholi MA-M, Gram C, Nielsen CAW, Ashina M (2020) Targeting BKCa channels in migraine: Rationale and perspectives. CNS Drugs 34:325–335. <https://doi.org/10.1007/s40263-020-00706-8> | BKCa channels are involved in CGRP and PACAP induced migraine pathways; BKCa channels are also coupled with K_ATP_ channels | | Yes |
|  | Clement A, Christensen SL, Jansen-Olesen I, et al (2023) The ATP sensitive potassium channel (KATP) is a novel target for migraine drug development. Frontiers in Molecular Neuroscience 16:1182515. <https://doi.org/10.3389/fnmol.2023.1182515> | K_IR_6.1/SUR_2_B antagonists are a potential treatment avenue for migraine | | Yes |
|  | Andres-Bilbe A, Castellanos A, Pujol-Coma A, et al (2020) The background K+ channel TRESK in sensory physiology and pain. Int J Mol Sci 21:5206. <https://doi.org/10.3390/ijms21155206> | TRESK frameshift mutation associated with migraine results in the transcription of an inhibitory molecule against TREK1 and TREK2, increasing the hyperexcitability of trigeminal neurons. | | Yes |
|  | Vincent M, Hadjikhani N (2007) The cerebellum and migraine. Headache: The Journal of Head and Face Pain 47:820–833. <https://doi.org/10.1111/j.1526-4610.2006.00715.x> | None | | No |
|  | Al-Karagholi MA-M, Hansen JM, Severinsen J, et al (2017) The KATP channel in migraine pathophysiology: a novel therapeutic target for migraine. J Headache Pain 18:90. <https://doi.org/10.1186/s10194-017-0800-8> | K_ATP_ openers induce headache like symptoms | | Yes |
|  | Al‐Karagholi MA, Hakbilen CC, Ashina M (2022) The role of high-conductance calcium-activated potassium channel in headache and migraine pathophysiology. Basic Clin Pharmacol Toxicol 131:347–354. <https://doi.org/10.1111/bcpt.13787> | BKCa opener MaxiPost precipitates migraine attacks | | Yes |
|  | Schreiber JA, Düfer M, Seebohm G (2022) The special one: Architecture, physiology and pharmacology of the TRESK channel. Cell Physiol Biochem 56:663–684. <https://doi.org/10.33594/000000589> | TRESK frameshift mutation is associated with migraineurs in a migraine pedigree | | Yes |
|  | Zhang Y, Wang H, Sun Y, et al (2023) Trace amine-associated receptor 1 regulation of Kv1.4 channels in trigeminal ganglion neurons contributes to nociceptive behaviors. J Headache Pain 24:49. <https://doi.org/10.1186/s10194-023-01582-5> | None | | No |
|  | Ávalos Prado P, Landra-Willm A, Verkest C, et al (2021) TREK channel activation suppresses migraine pain phenotype. iScience 24:102961. <https://doi.org/10.1016/j.isci.2021.102961> | TREK1 and TREK2 activation efficiently reverses NO-induced migraine-like phenotype | | Yes |
|  | Guo Z, Qiu C-S, Jiang X, et al (2019) TRESK K+ channel activity regulates trigeminal nociception and headache. eNeuro 6:ENEURO.0236-19.2019. <https://doi.org/10.1523/ENEURO.0236-19.2019> | Loss of functional TRESK protein greatly increases the likelihood of developing headache | | Yes |
|  | Mathie A, Veale EL, Cunningham KP, et al (2021) Two-pore domain potassium channels as drug targets: Anesthesia and beyond. Annu Rev Pharmacol Toxicol 61:401–420. <https://doi.org/10.1146/annurev-pharmtox-030920-111536> | TREK1, TREK2, TASK2, and TRESK are involved in nociception and migraine phenotype | | Yes |
|  | Mathie A, Veale EL (2015) Two-pore domain potassium channels: potential therapeutic targets for the treatment of pain. Pflugers Arch 467:931–943. <https://doi.org/10.1007/s00424-014-1655-3> | None | | No |
|  | Royal P, Ávalos Prado P, Wdziekonski B, Sandoz G (2019) [two-pore-domain potassium channels and molecular mechanisms underlying migraine]. Biol Aujourdhui 213:51–57. <https://doi.org/10.1051/jbio/2019020> | TRESK frameshift mutation associated with migraine results in the transcription of an inhibitory molecule against TREK1 and TREK2, increasing the hyperexcitability of trigeminal neurons. | | Yes |
|  | Cox HC, Lea RA, Bellis C, et al (2011) Variants in the human potassium channel gene (KCNN3) are associated with migraine in a high risk genetic isolate. J Headache Pain 12:603–608. <https://doi.org/10.1007/s10194-011-0392-7> | KCNN3 variants are associated with migraine in certain pedigrees | | Yes |
|  | Cannon SC (2001) Voltage-gated ion channelopathies of the nervous system. Clinical Neuroscience Research 1:104–117. <https://doi.org/10.1016/S1566-2772(00)00011-6> | None | | No |
|  | Lehmann-Horn F, Jurkat-Rott K (1999) Voltage-gated ion channels and hereditary disease. Physiol Rev 79:1317–1372. <https://doi.org/10.1152/physrev.1999.79.4.1317> | None | | No |
|  | Bourdain F, Fontaine B (2005) Voltage-gating ionic channels and neuromuscular disorders. EMC - Neurologie 2:403–429. <https://doi.org/10.1016/j.emcn.2005.07.004> | None | | No |
|  | Benarroch E (2022) What is the role of 2-Pore domain potassium channels (K2P) in pain? Neurology 99:516–521. <https://doi.org/10.1212/WNL.0000000000201197> | None | | No |
